# Beyond kill or no-kill: Institutional analysis of lethal control decision-making in large carnivore management

**DOI:** 10.1007/s13280-025-02317-3

**Published:** 2025-12-11

**Authors:** Nimisha Srivastava, John D. C. Linnell, Ramesh Krishnamurthy, Hannes J. Koenig, Christine Fürst

**Affiliations:** 1 Department Sustainable Landscape Development, Martin Luther University Halle-Wittenberg, Raum H4 1.41, Von Seckendorff-Platz 4, 06120 Halle (Saale), Saxony-Anhalt Germany; 2 Norwegian Institute for Nature Research, Trondheim, Norway; 3 Department of Forestry and Wildlife Management, University of Inland Norway, Postboks 400 Vestad, 2418 Elverum, Norway; 4 Department Landscape Level Planning and Management, Wildlife Institute of India, Post Box #18, Chandrabani, Dehradun, Uttarakhand 248001 India; 5 Department of Forest and Conservation Sciences, University of British Columbia, Vancouver, Canada; 6 Thaer-Institute of Agricultural and Horticultural Sciences, Humboldt Universität zu Berlin, Rudower Chaussee 16, 12489 Berlin, Germany; 7 German Centre for Integrative Biodiversity Research (iDiv) Halle-Jena-Leipzig, Raum H4 1.22, Von Seckendorff-Platz 4, 06120 Halle (Saale), Saxony-Anhalt Germany

**Keywords:** Carnivore management, Comparative conservation policy, Decision-making, IAD framework, Protected species derogation, Sociopolitical processes

## Abstract

Countries use lethal control as a tool to respond to human–large carnivore conflicts to varying degrees. The aim of this study was to explore the complexities surrounding the often-controversial decision to lethally manage carnivores. We examined the cases of the tiger (*Panthera tigris*) in India and the wolf (*Canis lupus*) in Germany. This study used an Institutional Analysis and Development framework to analyze contrasting sociopolitical processes. Through a review of legislative documents (*n* = 44) and interviews with experts (*n* = 47), the study examined the intricacies and challenges of the decision-making process and its implementation. While both countries were restrictive in their use of lethal control, decisions were primarily shaped by culturally embedded tolerance thresholds, accountability structures of decision-makers and influential societal factors. The findings demonstrate that effective carnivore management requires careful institutional design balancing scientific evidence with democratic participation.

**Graphical abstract:**

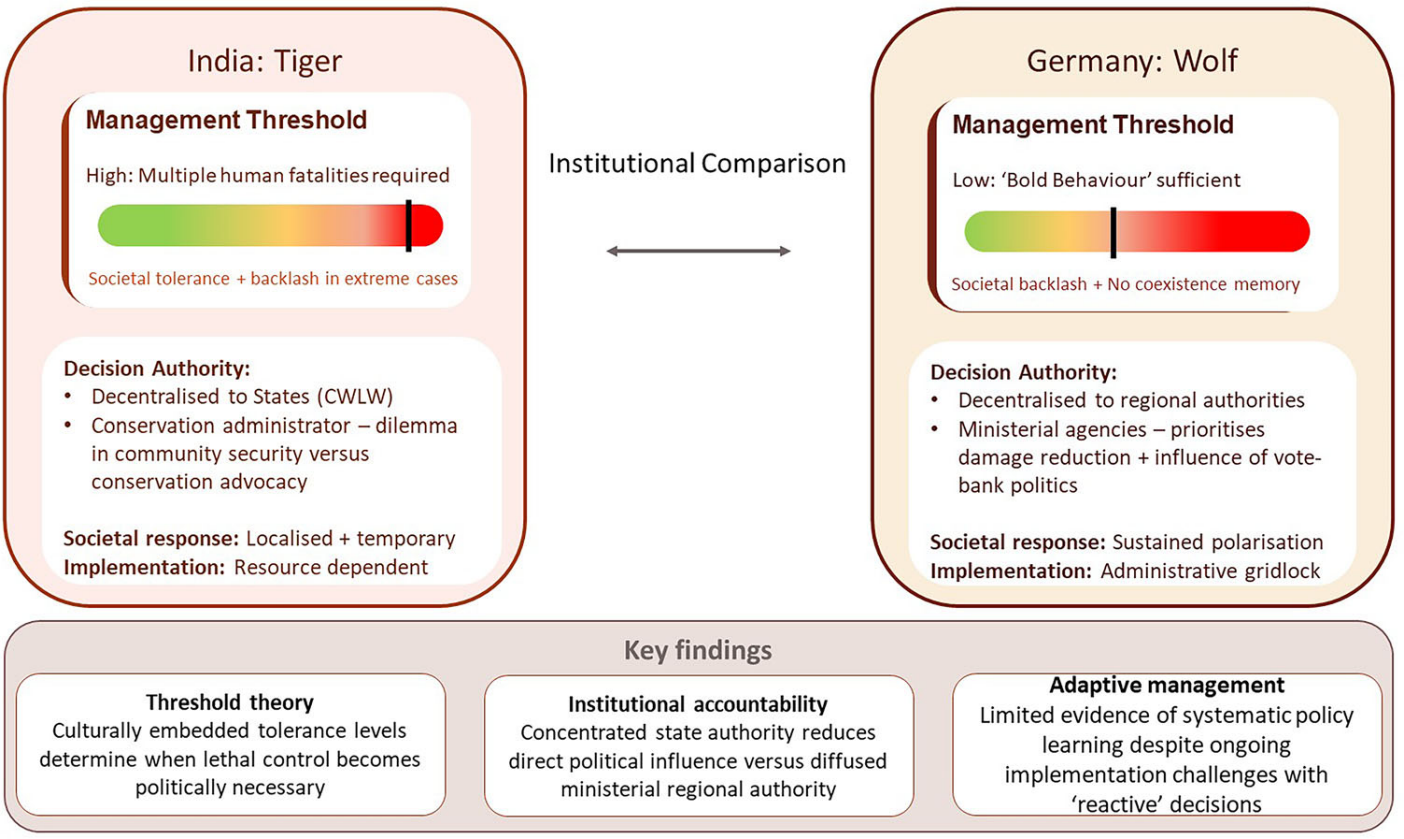

**Supplementary Information:**

The online version contains supplementary material available at 10.1007/s13280-025-02317-3.

## Introduction

Managing human–large carnivore conflicts is a major challenge for wildlife conservation in the light of the need to consider both conservation outcomes and social justice (König et al. 2020). The human–carnivore coexistence agenda, however, serves as the basis for management of negative interactions between people and carnivores. Following Carter and Linnell (2016, 2023), “coexistence” can be understood as a dynamic state in which humans and carnivores continuously co-adapt to each other’s presence. This should be supported with governance mechanisms that allow humans to minimize, and better face, the potential negative effects of carnivores, such as livestock depredation and in extreme cases, human deaths. There are two general strategies that countries employ to deal with human–carnivore interactions, nonlethal and lethal measures (Lorand et al. 2022). Nonlethal measures include compensation and insurance schemes, installing preventive measures such as livestock guard dogs and/or fencing livestock farms (Köck 2019; Ahmed and Khan 2022). Lethal measures are used in cases when an individual carnivore represents a potential danger for human lives and/or property (Linnell et al. 2017), or where populations can be managed as a game species and subjected to sustainable hunter harvest to regulate their population sizes (Cusack et al. 2022).

For example, some countries proactively use lethal control to regulate the size, and distribution, of large carnivore populations, for example, in Norway, Finland, Estonia and some states of the USA (Linnell et al. 2017; Mech 2017). Others selectively use lethal measures on a case-by-case basis, for example, when a carnivore becomes potentially “dangerous” to human lives and/or property. However, lethal measures are often controversial and can be heavily criticized nationally and internationally on both scientific and ethical grounds (Epstein 2017; Lute et al. 2018; Trouwborst and Fleurke 2019). Despite the debates around issues of lethal action taken against “problem” individuals, little is known about the procedural nuances of when countries deem it acceptable to kill a carnivore (Kaltenborn and Linnell 2022). This is particularly interesting in democratic countries that need to align peoples’ desires with conservation goals (Redpath et al. 2017). This can be complicated given the multi-level nature of modern democracies where legislation is often nested in a hierarchical structure of administrative units ranging from the local to the international.

In this study, taking tiger (*Panthera tigris*) and wolf (*Canis lupus*) as focus species, we explore how carnivore conservation policies under protected status unfold in different societies and investigate the guiding mechanisms behind such decisions. This study uses India and Germany as a case studies to compare complexities of sociocultural contexts and political frameworks in relation to the use of lethal measures to mitigate negative human–carnivore interactions.

### Why compare two different settings?

A comparative approach between two socioculturally distinct countries provides valuable insights into similarities and differences in addressing extreme conflict situations’ that would otherwise hamper the conservation goals of a country (de Boon et al. 2021). The focuses on India and Germany as case studies allow an exploration of the factors influencing national decisions to derogate from carnivore protection, and the varying societal responses to such derogations.

This research is based on the understanding that cultural attitudes toward nature vary across societies, shaping differing levels of tolerance toward carnivores. India’s case of tiger presents encounters between human and tiger that often lead to extreme conflict situations, such as human death(s) (Bombieri et al. 2023). However, cultural and religious significance of tigers along with strict protection laws reflect tolerance and coexistence with tigers (Oommen 2021; Jhala et al. 2021). Germany, on the other hand, demonstrates a perplexed situation where the recent resurgence of wolf populations has led to divided perspectives in society largely in the narratives of left versus right-wing ideologies and along the urban–rural divide (Pates and Leser 2021). Therefore, the aim of this study is to examine how India and Germany navigate the use of “lethal measures” in dealing with human–carnivore conflicts, specifically addressing the circumstances under which it is deemed acceptable to kill protected large carnivores. This study hypothesizes that the binary debate on “kill versus don’t kill” is shaped by nuanced approaches, influenced by sociocultural and political inclinations toward a carnivore. In “Decision-making processes for lethal control” and “The role of actors in the lethal control decisions” section, this study provides details on how the two countries differ in sociocultural, economic and political aspects. Use of lethal methods as a way to mitigate human–carnivore conflicts is a highly controversial topic that receives support as well as backlash from different segments of society (Linnell et al. 2017; Lute et al. 2018). Furthermore, variations in societal acceptance of negative human–carnivore interactions offer valuable insights into national responses to extreme situations. Such comparisons provide an overview of different possibilities of when killing a carnivore becomes acceptable. For instance, while the Indian subcontinent is recognized for its wildlife tolerance, it also experiences high incidences of carnivore attacks on humans (Bombieri et al. 2023). Conversely, in Germany, wolf attacks on livestock and perceived threats to human safety serve as triggers for rural populations, including stakeholders like hunters and shepherds, to advocate for relaxation in wolf-killing restrictions (Niedziałkowski 2022).

### Why we use tiger and wolf as examples?

India is home to several large carnivores, including big cats, bears and wild canids, where “Project Tiger” promotes the tiger as an umbrella species for the conservation of co-predators and prey populations (Qureshi et al. 2023). A strict protection status is given to all co-predators including Indian wolf (*Canis lupus pallipes*), common leopard (*Panthera pardus*), sloth bear (*Melursus ursinus*) and Himalayan black bear (*Ursus thibetanus*) that are in frequent and direct conflicts to humans (fatal attacks on humans) (Bombieri et al. 2023) under the Wildlife Protection Act, 1972 and the subsequent amendments in 2006 and 2023 (Srivathsa et al. 2022). Tiger is one of the predators that attacks humans either due to chance encounters or in predation events (Bombieri et al. 2023).

Germany hosts populations of wolves and Eurasian lynx (*Lynx lynx*), and a single individual brown bear (*Ursus arctos*) has visited in the last two decades (Kaczensky et al. 2024). Wolves are steadily increasing and are in conflict with people, mostly due to livestock depredation, perceived threat to humans and competition for game animals (Stohr and Coimbra 2013). At present, there are no significant direct threats from wolves to human lives (Linnell et al. 2021). Recent amendments to the Bern Convention and Habitats Directive have reduced the wolf's protection status from 'strictly protected' to 'protected' (European Parliament and the Council 2025). Member states, such as Germany, are currently deliberating on the implementation of this modified protection status within their national legal frameworks. Both the species are at the center of conservation policies framed around the management of human–carnivore conflict.

Accordingly, our research questions are (1) how do sociocultural and political factors influence the choice of dealing with carnivores in conflict, especially by taking lethal measures in different sociocultural and political contexts, and (2) what are the underlying rationales shaping the use of these measures?

### Theoretical and analytical framework

**Fig. 1:**
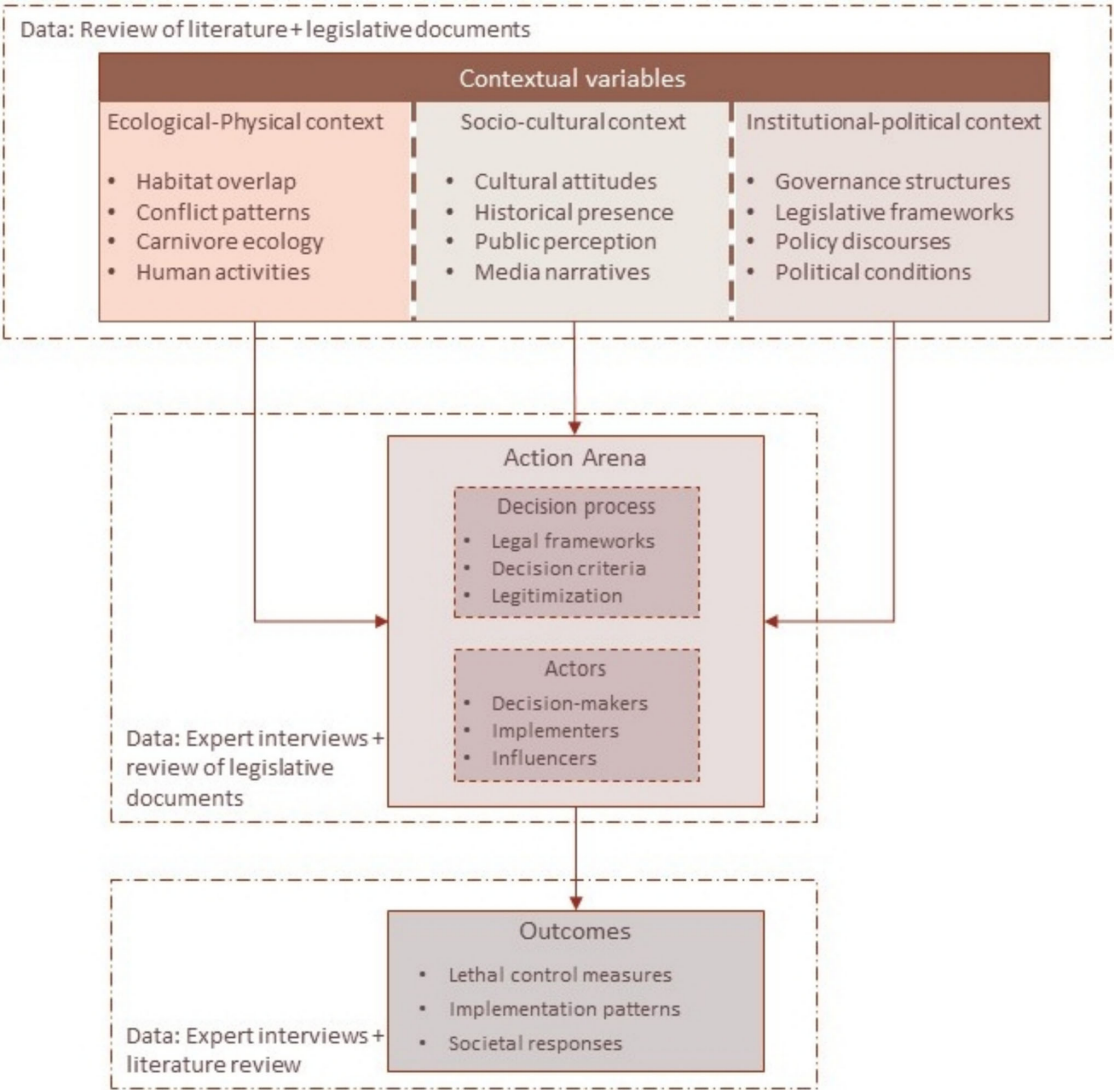
Adapted IAD framework used in our study to determine the role of contextual variables in the lethal control decisions to manage human–carnivore conflict. The action arena constitutes the decision-making process, including formal and informal rules, and the challenges with their implementation. It also examines the role of formal and informal actors in the decision-making process. Finally, interactions between different set of variables determine the outcomes of decisions. We referred to different data sources using a mix-method approach

We used the “heterologous horizontal axis” comparative approach sensu Bartlett and Varvus (2017), due to the contrasting sociocultural and legislative practices in India and Germany. The method is used in comparative case studies where the phenomenon that is to be studied remains the same, but may have differences due to different sociocultural ethnographies. Horizontal comparison requires careful examination of norms in a society that have shaped the cases differently. Hence, this allows comparing different cases without imposing or flattening cases to fit each other (Bartlett and Vavrus 2016). Taking this approach as a basis, we use the Institutional Analysis and Development (IAD) Framework (Ostrom et al. 1994; Ostrom 2005). The IAD framework can be used as a tool for analyzing governance complexities across diverse contexts. The framework has been used in different policy and institutional settings in the field of natural resources management, for example, in the context of forest governance (Nyaupane et al. 2022) or sustainable water governance (Bilalova et al. 2025). We have adapted the framework to fit to our case study (Fig. 1) where we define “contextual variables” as interactive group of variables that affect the “action arena” to produce certain “outcomes.” The variables include ecological and physical contexts, the sociocultural context as well as the institutional and political context influencing human–carnivore conflicts in each case study. An action arena in our case is the ground of a conflict situation, which is an interplay of the actors involved and the decision-making process that finally guides the outcome.

## Material and methods

### Study area

India and Germany represent strategically contrasting contexts for examining lethal control decision-making in carnivore management. These nations embody distinct positions along multiple comparative dimensions relevant to the research questions. India, with its centuries-long history of tiger coexistence, traditional cultural reverence for wildlife, and postcolonial conservation paradigm, contrasts sharply with Germany's recent wolf recolonization following historical extirpation, utilitarian wildlife traditions, and EU-driven policy framework (Chapron and Lopez-Bao 2014; Jhala et al. 2021; Di Bernardi et al. 2025). Additionally, the continuous versus interrupted history of carnivore presence offers insights into how experience shapes institutional responses and public attitudes toward conflict management.

Furthermore, these countries both represent different models of a federal structure (Jeffery et al. 2014; Arora and Kailash 2018), with very different conservation approaches (protected area-centric versus landscape integration), and socioeconomic contexts (developing versus developed economy) (Jhala et al. 2025; Di Bernardi et al. 2025). Their different stages in the intensities of human–carnivore conflicts and variations in stakeholder perspectives facilitates analysis of how diverse sociopolitical factors influence lethal control decisions across contexts.

This comparative framework allows us to isolate the influence of sociocultural and political variables on decision-making processes while controlling for the biological similarities of apex predator management challenges, thereby addressing our research questions regarding the drivers and rationales behind lethal control implementation.

### Data collection

We followed a mix-method approach of literature review, expert interviews and review of legislative documents relevant to the research objective of the study. We conducted purposive narrative literature review (Rother 2007). Peer-reviewed articles were selected based on the topic. This included, literature specific to the conflict patterns (attack cases on livestock and humans) of tigers in India and wolves in Germany; and second, human perceptions on the study species in each case study.

Expert interviews were conducted with 47 participants (25 in India and 22 in Germany) between February 2022 and January 2025, with an average duration of 55 min. Initial expert identification employed purposive sampling by the authors, followed by snowball sampling based on expert recommendations to ensure institutional diversity and reach data saturation. The final sample represented diverse professional backgrounds: administrators (30%), stakeholder representatives (20%), conservationists (18%), policymakers (15%), social scientists (11%), and NGOs (6%). Given the exploratory nature of this research, follow-up interviews were conducted with selected participants to deepen understanding of governance challenges in carnivore conservation and human–carnivore interaction management. The iterative sampling process included four additional experts—three in India (two stakeholder representatives and one researcher) and one state administrator in Germany—all of whom are included in the total sample of 47. This approach ensured comprehensive representation of institutional diversity while achieving theoretical saturation. Interview guidelines are available in supplementary material S1.

Qualitative interview data were analyzed using Braun and Clarke's (2006) six-phase thematic analysis approach within a qualitative content analysis framework. The analysis progressed systematically from data familiarization and initial line-by-line coding through pattern identification to theme development and refinement. The coding process was conducted inductively, allowing themes to emerge from the data rather than being imposed by predetermined theoretical frameworks. Constant comparative analysis was employed throughout the process to ensure consistency and reliability in theme development. All coding was performed using MaxQDA software (VERBI software 2021) with regular peer debriefing sessions conducted to enhance analytical rigor and reduce researcher bias.

The final thematic framework comprised seven major themes: conflict scenarios, governance structure, governance challenges, actors in the action arena, implementation processes, implementation challenges, and recommendations. Subthemes were identified within the broader thematic categories to capture the nuanced variations within themes (see supplementary material S2). Data saturation was achieved when no new themes or significant variations emerged from the continued analysis of interview transcripts.

We complemented our findings with a qualitative review of legislative documents to understand national policies on carnivore management, specifically regarding lethal control decisions. The selection of documents was based on the authors' prior knowledge, expert recommendations, and an iterative reading process that allowed for the inclusion of additional relevant materials. The documents included laws, guidelines, standard operating procedures, court cases, such as from state high courts in both countries and the Supreme Court of India and the Court of Justice of the European Union (CJEU) that are directly or indirectly related to managing human–carnivore conflicts in both case studies (supplementary material S3). While this approach does not follow a formal systematic review framework, it ensures a comprehensive and contextually grounded understanding of the legal landscape governing carnivore conservation and management. A total of 44 legislative documents (19 from India and 25 from Germany) were reviewed that were selected based on their relevance to the research objectives. Documents with original text in German were translated using Google translate. India has a quasi-federal system where national laws and policies guide tiger conservation and management in the country. These laws are followed and implemented by the state forest department across different protected areas (Srivastava et al. 2025). Because Germany has a federal system, we targeted two states in Germany, the state of Lower Saxony and the Brandenburg state. Both states host highest densities of wolves (DBBW 2025). The federal state of Germany is also embedded in the European Union which has a strong wildlife conservation legislation.

## Results

### Contextual influences on lethal control decision-making

#### Ecological–physical context: Shared habitats and conflict interfaces

Both India and Germany feature human-modified landscapes characterized by forest fragments interspersed within agricultural matrices (Jhala et al. 2025; König et al. 2023). These shared habitats create extensive human–carnivore interfaces where conflicts emerge. In India, approximately 30% of the tiger population (~ 4000 individuals) utilizes territorial forest land outside protected areas (Qureshi et al. 2023). While some individuals also use agricultural landscapes for dispersal or as seasonal habitats (Warrier et al. 2020). Similarly, wolves in Germany, which have been recolonizing since 2000, predominantly occupy multi-use, non-protected habitats within human-dominated landscapes (Jarausch et al. 2021; Kiffner et al. 2022). While they show a general preference toward patches of undisturbed habitat over agricultural landscapes (Planillo et al. 2024) they are almost totally dependent on multi-use, non-protected habitats in the human-altered landscapes of Germany (Khorozyan and Heurich 2022).

The high human population densities—485 people/km^2^ in India and 238 people/km^2^ in Germany (World Population Review 2024) coupled with development priorities and settlement patterns, intensify the human–carnivore interface. In India, urban and agricultural expansion alongside increasing tiger populations has blurred boundaries between forests and human settlements, escalating conflict incidents. This is coupled with dependence on forest resources as a source of livelihood, for fodder and fuelwood, which increases chances of human encounters with tigers (Soman and Anitha 2020; Ahmed and Khan 2022). Due to increasing urbanization and unavailability of “grazing zones,” unprotected livestock grazing is reported from different protected areas across the country (Shukla et al. 2025).

In Germany, the rapid increase in wolf populations (from total absence to over 1,000 individuals within two decades) has created conflict interfaces particularly in areas of recent recolonization, where livestock keepers lack preparedness or where wolves have overcome preventive measures (König et al. 2023). Germany’s cultural landscapes largely consists of private and public forest patches interspersed within agricultural landscapes, often comprising of protected/unprotected livestock farms.

These shared habitats and conflict interfaces are embedded within distinct sociocultural contexts, with India's traditional tolerance toward carnivores contrasting with Germany's historical perception of carnivores as pests which led to their extermination, though contemporary attitudes in both countries reflect complex and evolving human–carnivore relationships that we discuss in the next section.

#### Sociocultural context: Divergent cultural attitudes and historical relationships

Indian and German societies exhibit distinct sociocultural frameworks regarding carnivore coexistence, manifested through different belief systems and cultural symbolism. In India, human–carnivore relationships are characterized by complex cultural integration, with carnivores occupying significant positions in religious narratives, mythology, and folklore (Oommen 2021). This cultural embeddedness has fostered traditional tolerance toward tigers despite their potential threats to human safety (Jolly et al. 2022; Beggiora and Exley 2024). Despite hardships caused by conservation policies, local communities often express support for the forest department due to internalized state narratives, cultural acceptance of authority, and psychological coping mechanisms like system justification (Martin et al. 2018). However, recent landscape transformations through urban expansion, agricultural intensification, and increasing tiger populations have created novel conflict interfaces, exceeding established tolerance thresholds in some communities. Consequently, when conflict intensity escalates—particularly following multiple tiger attacks—communities increasingly advocate for lethal management interventions by the Forest Department (Bhardwaj 2021; Deshpande 2023).

In contrast, European and specifically German historical perspectives framed large carnivores as antagonistic entities warranting systematic eradication, resulting in regional extirpation of wolf populations by the early twentieth century (Boitani and Linnell 2015). Contemporary German society exhibits polarized attitudes toward naturally recolonizing wolves (Arbieu et al. 2019; Zscheischler and Friedrich 2022), with discernible divisions along urban–rural and political-ideological gradients (Pates and Leser 2021). Urban populations predominantly conceptualize carnivores as positive wilderness indicators, while rural communities prioritize concerns regarding livestock depredation and human safety, thereby influencing management policy discourse and derogation frameworks (Kiffner et al. 2019). The accelerated population recovery—from functional absence to exceeding 1000 individuals within two decades—has intensified social conflicts, particularly manifesting as livestock depredation incidents in regions of recent wolf recolonization where preventive measures remain inadequately implemented or have been circumvented, especially among small-scale and semi-professional livestock operations (Ostermann-Miyashita et al. 2025).

#### Institutional–political context: Multi-level governance and political significance

Tiger and wolf conservation governance in India and Germany, respectively, represent distinct institutional approaches within contrasting political frameworks, reflecting different conservation paradigms and administrative structures.

India employs a predominantly land-sparing approach to tiger conservation within its quasi-federal governance structure, where conservation authority is distributed between central and state governments under the Constitutional Amendment of 1976. The Wildlife Protection Act (1972) established the overarching legal framework, while Project Tiger (1973) institutionalized conservation efforts following alarming population declines from approximately 40,000 precolonial individuals to merely 3500 by 1972 (Panwar 1982; Jhala et al. 2021). Contemporary tiger conservation administration operates through a hierarchical structure with the National Tiger Conservation Authority (NTCA) providing policy directives and state Forest Departments implementing and planning territorial management. While protected areas represent the cornerstone of tiger conservation, approximately 30% of India's tiger population utilizes multi-use landscapes, necessitating coexistence strategies (Qureshi et al. 2023). Conservation policies incorporate conflict mitigation through ex-gratia compensation schemes and initiatives aimed at reducing forest resource dependency, thereby minimizing human–tiger encounters (Jhala et al. 2025). Economic benefits generated through tourism are shared with local communities through programs such as eco-development committees (EDCs). This measure aims to gain trust, legitimacy and support for conservation from rural communities. Human–tiger conflict incidences remain politically localized, with controversies typically emerging around specific conflict incidents rather than generating systemic national debate (Rastogi et al. 2014).

Germany exemplifies a land-sharing approach within its federal political system comprising 16 states. Wolf protection emerged during the species' regional absence through international instruments—the Bern Convention (1979) and subsequently the EU Habitats Directive (1992)—which designated wolves as strictly protected under Annexes II and IV (Reinhardt et al. 2013; Niedziałkowski 2022). However, in March 2025, wolf’s protection status was down-listed to “protected” in Appendix III of the Bern Convention and Annex V of the Habitats Directive (Council of Europe 2025; EU Commission 2025). This was a long-standing proposal from the Member States to the EU Parliament. The reduced protection status is aimed to allow an ease in lethal control regulations, which have often been challenged at the Court of Justice of the European Union (CJEU). However, despite reduced protection status, management practices should not negatively impact the conservation goal of the species. This is until wolf population reaches a “favorable conservation status” (FCS) (Trouwborst et al. 2017). However, the definition of FCS is unclear which allows misinterpretations and controversies around decisions to legally kill a problem wolf, or a wolf pack (Epstein et al. 2015, 2019). However, a group of NGOs has taken the amendment decisions for both, the Bern Convention and the Habitats Directive to the CJEU, challenging its legitimacy as it contradicts EU’s environmental legislation that a strictly protected species cannot be managed at population levels until it has reached an FCS (Köck 2019; Guillot 2025). When wolves were strictly protected under the Bern Convention/Habitats Directive, it was only possible for Germany to issue permits to kill wolves under a set of clearly defined conditions. In practice, Germany exercised a far more restrictive application of this jurisdictional discretion than many other countries, so the impact of the EU level strict protection served as only an indirect constraint on German decisions (Linnell et al. 2017). Despite the change in wolf protection status, the Federal Nature Conservation Act currently remains unmodified (October, 2025). However, proposals to manage wolves as a huntable species under Federal hunting law are under consideration.

The administrative responsibility for wolf management resides primarily with state environmental agencies, creating spatial heterogeneity in monitoring protocols and management interventions. Unlike India's integrated conservation-forestry administration, German wolf management operates through institutional arrangements involving both environmental and agricultural authorities. These institutions implement different approaches to conflict mitigation, including substantial preventive measures (electric fencing subsidies, livestock guarding dogs) and compensation schemes (Brandenburg 2019; Lower Saxony 2022). Wolf conservation has evolved into a politically divisive issue, with polarization along rural–urban and ideological dimensions, manifesting in party politics ranging from advocating strict protection to proposing down-listing to Annex V for hunting management (Pates and Leser 2021). This politicization has elevated wolf management beyond ecological consideration to a symbolic proxy for broader sociopolitical tensions in contemporary German society. Table 1 provides a summary of contextual variables for the case studies.

**Table 1:** A summary of the contextual variables in the case study countries influence the decision-making and implementation processes surrounding decisions on lethal management of carnivore to manage human–carnivore conflict situation

Ecological–physical context	***Landscape feature***	Protected forests interspersed with agriculture and settlements	Fragmented forest patches within agricultural and seminatural landscapes	König et al. (2023), Jhala et al. (2025)
	***Human population***	Human population density is estimated at around 480 people / km^2^. With 62.92% of the population inhabiting rural areas	Human population density is estimated at around 240 people / km^2^. Around 23.50% of the population inhabits rural areas	World Population Review (2024), Worldometers.info (2025)
	***Carnivore population***	India holds 75% of global tiger population with around 3682 tigers in 18 out of 28 states and 8 union territories	Germany is home to around 1157 wolf individuals with 161 wolf packs, 42 pairs, and 21 single residents in 10 out of 16 federal states	Qureshi et al. (2023), DBBW (2025)
	***Carnivore population trend***	Increasing within reserves, stable or declining outside the reserves	Increasing and expanding into new regions	Qureshi et al. (2023), DBBW (2025)
	***Human–carnivore conflict patterns***	Rural communities, including forest-dwelling tribes, in India are dependent on forests for timber, fodder and livelihoods. Along with this, livestock grazing within and around protected areas is a common practice. This increases probabilities for direct encounters with tigers, often leading to human fatalities. In some regions, tigers actively hunt humans for meat, whereby they are declared “dangerous to human lives” and are either captured or killed. Depleting forest habitats and increasing land-use changes are attributed to increasing human–tiger conflicts	In Germany, human–wolf conflict is largely due to livestock depredation, and perceived threat to human lives. Due to regional differences, personal choices of livestock herders, and purpose of livestock herding (such as sheep/goats used to manage *deich*), they may or may not be protected with electric fencing or guard dogs. Wolves using agricultural areas often predate on livestock, both protected and unprotected. However, if there are repeated cases of wolf hunting protected sheep they are authorized to be killed	Kiffner et al. (2022), König et al. (2023), DBBW (2025), Jhala et al. (2025),
			People also use forested areas for recreational purposes, presence of wolves in a region is often associated with perceived fear of threat to human lives. As of yet, there are no cases of wolf that has become dangerous to human lives in Germany	
	***Historical presence / absence***	Although tiger numbers declined drastically during the colonial era, their populations were never extinct from the country. This allowed a continuous sense of presence of tigers among local communities and hence adaptation to “living with tigers”	Wolves in Germany were extinct for around two centuries. This led to loss of traditional knowledge and experience of “living with wolves” in local communities	Reinhardt et al. (2013, Jhala et al. (2021, 2025) )
Sociocultural context	***Perceptions toward carnivores***	Tigers are associated with different cultural and religious significance across ethnicity within the country. These positive associations allow tolerance to some losses. However, increasing cases of livestock depredation and human fatalities may lead to shifts in perceptions	Wolves were historically attributed with negative perception and symbolism. However, contemporary urban population perceive wolves largely as positive, whereas rural populations perceive them largely as negative	Struebig et al. (2018), Arbieu et al. (2019), Zscheischler and Friedrich (2022), Chatterjee (2023)
Institutional–political context	***Carnivore protection status***	Tiger is strictly protected under the Wildlife (Protection) Act, 1972 and its subsequent amendments	Wolf is “protected” under the Bern Convention and the Habitats Directive following a recent amendment affective since June, 2025. However, conservation focus still aims for reaching a “favorable conservation status”	WPA (1972, 2002, 2006, 2023), EU Commission (2025), Council of Europe (2025), European Parliament and the Council (2025)
	***Management and administration***	State-run administration through central authority NTCA and the state forest department	State-run administration through Federal and state-level management	Jhala et al. (2021, 2025), Reinhardt et al. (2013)
	***Mitigation measures***	Preventive measure includes restrictive use in protected areas. Compensation for livestock loss and human loss to tiger	Livestock preventive measures include state-subsidized electric fencing and guarding dogs. Regional authorities compensate for livestock losses	Jhala et al. (2025), Kiffner et al. (2022)
	***Stakeholder participation***	Local communities are included within forest administration at local levels through joint forest management programs, such as eco-development committees by the state forest department following national laws	Collaborative policymaking designed to incorporate stakeholder representatives and NGOs for state wolf management plan	Das (2019), Zscheischler Busse and Heitepriem (2019)

### Decision-making processes for lethal control

#### Legal frameworks and their implementation: The gap between law and practice

In both India and Germany, wildlife protection legislation establishes the foundational legal architecture for carnivore management, though implementation varies considerably between jurisdictions. In India, the Wildlife Protection Act (1972) provides a nationally uniform framework that explicitly delineates conditions under which tigers may be declared “dangerous to human lives,” typically following multiple human fatalities (NTCA 2007, 2013). This designation is a prerequisite for lethal control authorization. However, states / region with a history of tigers predating humans may intensify resources to monitor and capture tiger [IN_024]. German states operate within the Federal Nature Conservation Act (BNatSchG), (2009) which transposes EU Habitats Directive protections, permitting wolf removals when individuals exhibit “bold” behavior (approaching humans within 30 m or repeatedly entering settlements) or demonstrate capacity to overcome standardized preventive measures. In March, 2025, the Bern Convention and therefore, the Habitats Directive have down-listed the protection status of wolves as “protected” (European Parliament and the Council 2025). The Member States, including Germany, are yet to define how and in what conditions they would further define wolf management. Therefore, our analysis is based on the existing framing of wolf management in Germany. More details on the legal provisions for derogation to protected status in both countries are provided in supplementary material S4. We also provide details on the working rules that inform the decision-making process and the outcomes in supplementary material S5.

Despite these established frameworks, significant implementation disparities emerge across regions in both nations. In India, resource inequities between economically advantaged and disadvantaged states directly impact operational capacity. One researcher [IN_025] highlighted: "*Junnar, an agricultural region near Mumbai with high leopard-human conflict incidence, receives substantial financial support for Rapid Response Teams and equipment, while Vidarbha, experiencing similar conflict levels, lacks comparable resources due to its distance from India's financial hub."* This disparity manifests in differential response capabilities and conflict resolution outcomes.

In Germany, implementation challenges center on procedural complexity and bureaucratic delays. After genetic confirmation of wolf responsibility in livestock depredation events—itself a time-consuming process—state environmental ministries must contract licensed hunters for targeted removals. As one farmers' association representative [DE_020] noted: "*The delay in hunting a problem animal is an issue because it is not easy to identify the responsible individual—can't do genetics through eyes. Also, it is hard to kill a wolf with a gun.*"

Both systems demonstrate reactive rather than proactive approaches to conflict management. As one Indian scientist [IN_010] observed: "*Preventive measures actively try to prevent situations. But in India, mostly reactive mode occurs. Officers normally wouldn't contact [scientists], but as soon as there's a bear or leopard in a village, they start calling, asking 'what to do, how to take the animal away’. They never discuss methods to prevent conflict from happening.*" Similarly, a German scientist [DE_004] remarked on institutional unpreparedness: "*In Brandenburg's early wolf colonization period, we asked the government to prepare their institutional structures, but they never realized it until wolves were everywhere.*”

#### The decision criteria: Information flows and evidence

Evidentiary standards and verification processes differ substantially between the two management systems. In India, tiger identification focuses on establishing individual responsibility for human fatalities through a combination of field evidence, pattern and genetic analysis, and expert assessment [IN_005; IN_007; IN_009]. The protocols are largely based on SoPs provided by the NTCA [IN_001; IN_007; IN_011]. States prepare protocols for the Rapid Response Teams (RRTs) that conduct investigations while simultaneously managing public safety and crowd control in crisis scenarios [IN_024]. However, response efficacy varies significantly with institutional capacity. Limited technical training—one forest department official [IN_009] reported merely "*10 days of shooting/hunting training*" for personnel—constrains effective implementation.

Unlike tigers that can be identified individually based on their stripe patterns, wolves need more technologically intensive verification protocols, particularly genetic analysis to confirm wolf responsibility in livestock depredation events. Germany also inspects if adequate preventive measures were properly implemented. This forensic approach creates a higher evidentiary burden but faces practical limitations in individual wolf identification. As interviewees noted, genetic confirmation of an attack does not readily translate to field identification of the responsible individual [DE_019; DE_020].

Experts across both countries identified significant evidentiary gaps requiring attention. Indian specialists advocated strengthening community inclusion in policy development [IN_003; IN_005; IN_010], implementing landscape-level conflict management planning [IN_014; IN_016; IN_017; IN_018; IN_019], and deploying technological early-warning systems to prevent dangerous encounters [IN_002; IN_007; IN_008]. German experts emphasized improved public education on coexistence strategies [DE_004; DE_007; DE_009; DE_017], noting that "*wolves were not part of people's lives for 200–300 years in Germany… there is no knowledge at all, there are no institutions, and that's a major challenge*" [DE_21].

#### Justification and legitimization: How decisions are explained and defended

Both nations employ distinct justification frameworks for lethal control decisions that reflect their institutional contexts and social priorities, with legal proceedings revealing how these frameworks operate under scrutiny.

In India, the case of tigress T1 (‘*Avni*’) demonstrates how authorities justify lethal control through procedural compliance with the WPA, 1972. When linked to 13 human fatalities in Maharashtra's Yavatmal district, T1 was declared “dangerous to human life” by the CWLW. The ensuing legal challenge in the Bombay High Court (Writ Petition No. 5792/2018, among others) resulted in the court upholding the shoot order while mandating attempts at tranquilization first. Subsequently, when appealed to the Supreme Court, the court refused to interfere with the shooting order on September 11, 2018, deferring to the forest department's determination of necessity while maintaining the tranquilization requirement. After T1's killing, the procedural legitimacy of the action was contested through contempt proceedings in the Bombay High Court, with wildlife activists alleging violations of court-ordered protocols. This litigation sequence illustrates how Indian authorities construct justifications around procedural adherence to established legal frameworks, focusing on individual tigers' specific behavior patterns rather than population-level management concerns. More details on the case are provided in Box 1 (“Societal responses and conflict escalation” section) that further exemplify the ethical and moral grounds of killing or not killing a tiger.

**Figure:**
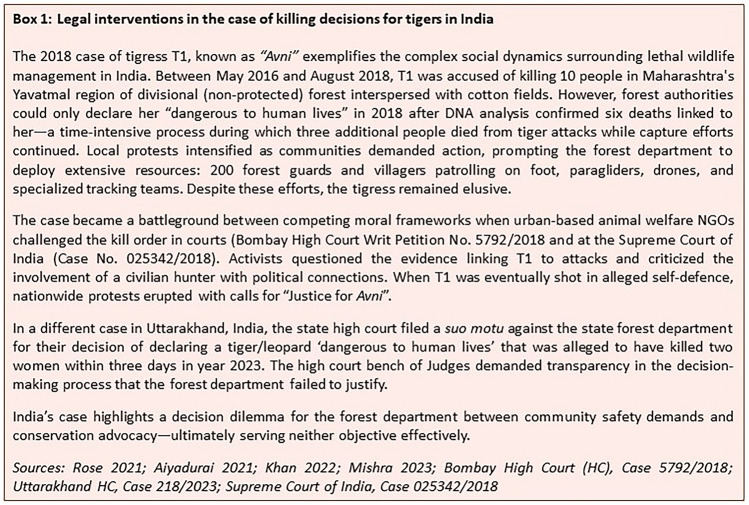

German authorities operate within the stringent constraints of EU environmental law, with Court of Justice of the European Union (CJEU) rulings progressively narrowing permissible justifications for wolf removal. The landmark ruling in Case C-601/22 (2024) and Case C-674/17 (2019) established three mandatory conditions for derogations as equal among all member states: absence of satisfactory alternatives, maintenance of favorable conservation status, and prevention of “serious damage.” Previous court rulings, such as Case C-342/05 (2007) mandated proof that lethal control would effectively prevent serious damage. The Case C-436/22 (2024) from Spain is relevant to the recent change of reduced wolf protection status across EU Member States. Wolf population in northern Spain are governed as Annex V species of the Habitats Directive. Despite the species being a “protected” species, the court rejected generalized claims about population management of wolves given that their population were still unfavorable.

Contrasting justification frameworks reveal fundamental differences in institutional constraints and underlying motivations. Indian authorities exercise greater discretional flexibility within national procedures when addressing legitimate human safety threats, enabling defensible administrative decisions. Conversely, EU member states navigate restrictive multilayered legal environments designed to prevent wildlife removal for potentially questionable political motivations. While structural constraints differ significantly, this disparity reflects distinct regulatory purposes: protecting human life versus safeguarding wildlife from illegitimate management pressures through sophisticated legal barriers.

### The role of actors in the lethal control decisions

#### Decision-making authority and accountability: Who decides and why

Decision-making authority structures reflect different governance arrangements in each country. In India, the Chief Wildlife Warden (CWLW) of each state holds singular authority for lethal control decisions, establishing clear accountability within the bureaucratic hierarchy. This centralized authority model operates within intense sociopolitical pressures during crisis situations. As one CWLW [IN_024] articulated: "*My decision on declaring a free-roaming tiger as 'dangerous to human life' would lead to its lifetime imprisonment in a zoo or killing. Hence, I need to know the situation details to be sure before making my decision.*"

The Indian decision-making process occurs amid complex stakeholder dynamics including forest department officials, administrative bodies [IN_001; IN_007], community protesters (often expressing discontent through demonstrations), and political figures exerting pressure for expedited action [IN_010; IN_025]. These situational pressures complicate objective decision-making and sometimes necessitate discreet management actions, as one forest official [IN_001] explained regarding the clandestine release of captured tigers to avoid public opposition.

Germany's authority structure is more diffuse, with responsibility distributed across state environmental ministries and subordinate agencies that function as decision-makers. Their determinations, however, exist within a highly politicized landscape characterized by polarized stakeholder positions. Conservation NGOs (WWF, NABU) and politically left-aligned parties advocate strict protection, while agricultural associations, hunting organizations, and right-leaning political entities push for management flexibility—creating what interviewees characterized as “pro-wolf” and “anti-wolf” coalitions [DE_001; DE_002; DE_010].

This polarization subjects German authorities to countervailing pressures and judicial oversight, as described by one state agency representative [DE_020]: "*Not all derogations made for wolf management are implemented because local NGOs take the decisions to court. Often the court rejects the authority's decision in cases where evidence to justify wolf killings are not sufficient*". Another state representative [DE_022] candidly acknowledged that "*everything around wolves is political*", highlighting how partisan considerations permeate ostensibly technical determinations.

#### Different equilibrium points in the decision-making

India and Germany demonstrate distinctive equilibrium points in balancing carnivore conservation with human interests, reflecting their socio-ecological contexts. In India, the equilibrium point heavily favors tiger conservation until tolerance thresholds are demonstrably breached through human fatalities. This conservation-centric equilibrium stems from tigers' cultural significance, their endangered status, and the national investment in tiger recovery since the 1970s. The threshold for lethal intervention remains high, requiring multiple human fatalities before a tiger may be declared “dangerous to human life.” This approach prioritizes the biological value of individual tigers within recovery efforts while acknowledging limits to social tolerance.

Germany's equilibrium point reflects greater sensitivity toward socioeconomic impacts, particularly agricultural interests, despite wolves' protected status. The threshold for intervention—wolves approaching human settlements or overcoming standard preventive measures—indicates greater consideration of human economic interests and perceived safety concerns. This equilibrium reflects the recent nature of wolf recovery, the absence of cultural adaptation mechanisms, and the political influence of agricultural stakeholders within German governance structures. Both equilibrium points represent contextually rational responses to distinct social, historical, and ecological conditions rather than universally optimal solutions.

#### Political pressures and public perception: Media and electoral dynamics

Political considerations significantly influence lethal control decisions in both contexts, though through different mechanisms. In India, political pressure typically manifests locally and reactively during acute conflict episodes [IN_001; IN_022]. Local politicians mobilize around specific incidents, particularly human fatalities, applying direct pressure on forest departments for immediate action [IN_022]. Media coverage amplifies community grievances, creating pressure for visible management responses.

German wolf management, conversely, has become emblematic of broader ideological divisions that transcend specific conflict incidents. The polarization between rural and urban constituencies, agricultural and conservation interests, and right-leaning versus left-leaning political formations transforms wolf management into proxy conflicts about rural autonomy, environmental governance, and cultural identity [DE_001; DE_002; DE_021]. Media narratives, as noted by stakeholders, often portray wolf incidents in sensationalized terms that reinforce existing polarization [DE_021]. Electoral considerations directly influence political positioning, with parties adopting increasingly entrenched positions on wolf management to appeal to their constituencies. This politicization transforms evidence-based management decisions into symbolic political acts, complicating rational policy development [DE_003; DE_011].

Media narratives substantially influence evidence perception and interpretation in both contexts, though more prominently in Germany. A shepherds' association [DE_015] representative observed that "*media portrays conflict incidents as though wolves are causing massacres on farms*," while an anthropologist [DE_021] noted that "*critical voices dominate public platforms, while supportive perspectives are scarce due to fears of backlash from rural populations*," creating an asymmetrical discourse environment.

### Outcomes of lethal control decision-making

**Fig. 2:**
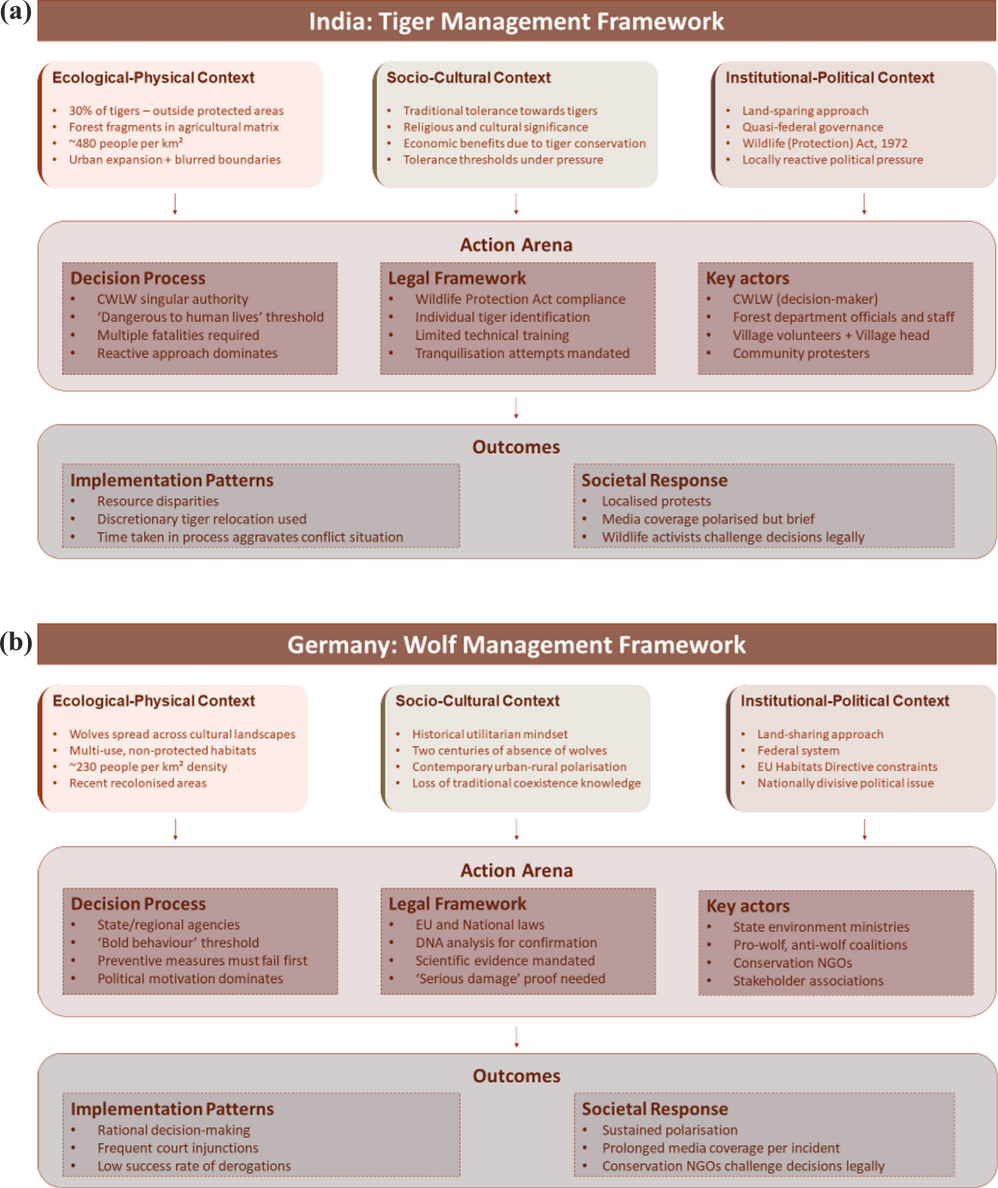
Carnivore management framework in India (**a**) and Germany (**b**). The framework illustrates how contextual variables (ecological–physical, sociocultural and institutional–political contexts) shape the action arena of lethal control decision-making, leading to specific implementation patterns and societal responses

The interplay between ecological–physical, sociocultural and institutional–political contexts fundamentally shapes the action arena, creating distinct pathways for decision-making processes that culminate in markedly different implementation patterns and societal responses. These contextual variables collectively determine both the operational feasibility of lethal control measures and the legitimacy frameworks through which such decisions are contested or accepted. Figure 2a, b demonstrates the interactions between these variables and how they provide different outcomes in both contexts.

#### Implementation patterns

Implementation patterns reveal fundamentally different operational challenges rooted in evidence requirements and resource constraints. In India, forest departments must distinguish between predatory events and chance encounters while managing high numbers of dispersing individuals. Officials regularly capture and relocate tigers from human settlements, but face complex decisions when animals return—particularly when rare attacks occur after release [In_001]. The evidentiary burden for declaring individuals “dangerous to human life” creates critical time delays: supporting evidence collection can span years while conflict escalates (Box 1), as departments balance legal requirements against mounting casualties and community pressure. Resource inadequacies compound these challenges, forcing reactive responses to crisis situations rather than systematic preventive management. Decisions on lethal management of tiger(s) create moral dilemma for the forest department (the decision authority) between immediate community safety needs and conservation advocacy.

German implementation confronts a different complexity: navigating restrictive legal frameworks while addressing political pressures. Despite comprehensive legal mechanisms, member states systematically attempt to exploit perceived loopholes in EU environmental law to satisfy electoral constituencies. State agencies operate within constraints where scientific evidence requirements conflict with political expectations for swift action. This creates a paradox where rational decision-making processes become tools for circumventing rather than implementing protective legislation (Box 2). The result is implementation gridlock: extensive administrative effort produces minimal actual management outcomes; as legal challenges consistently prevent authorized actions from proceeding. Unlike India's evidence-gathering delays, German delays stem from legal obstruction that in practice prevent rather than enable management interventions.
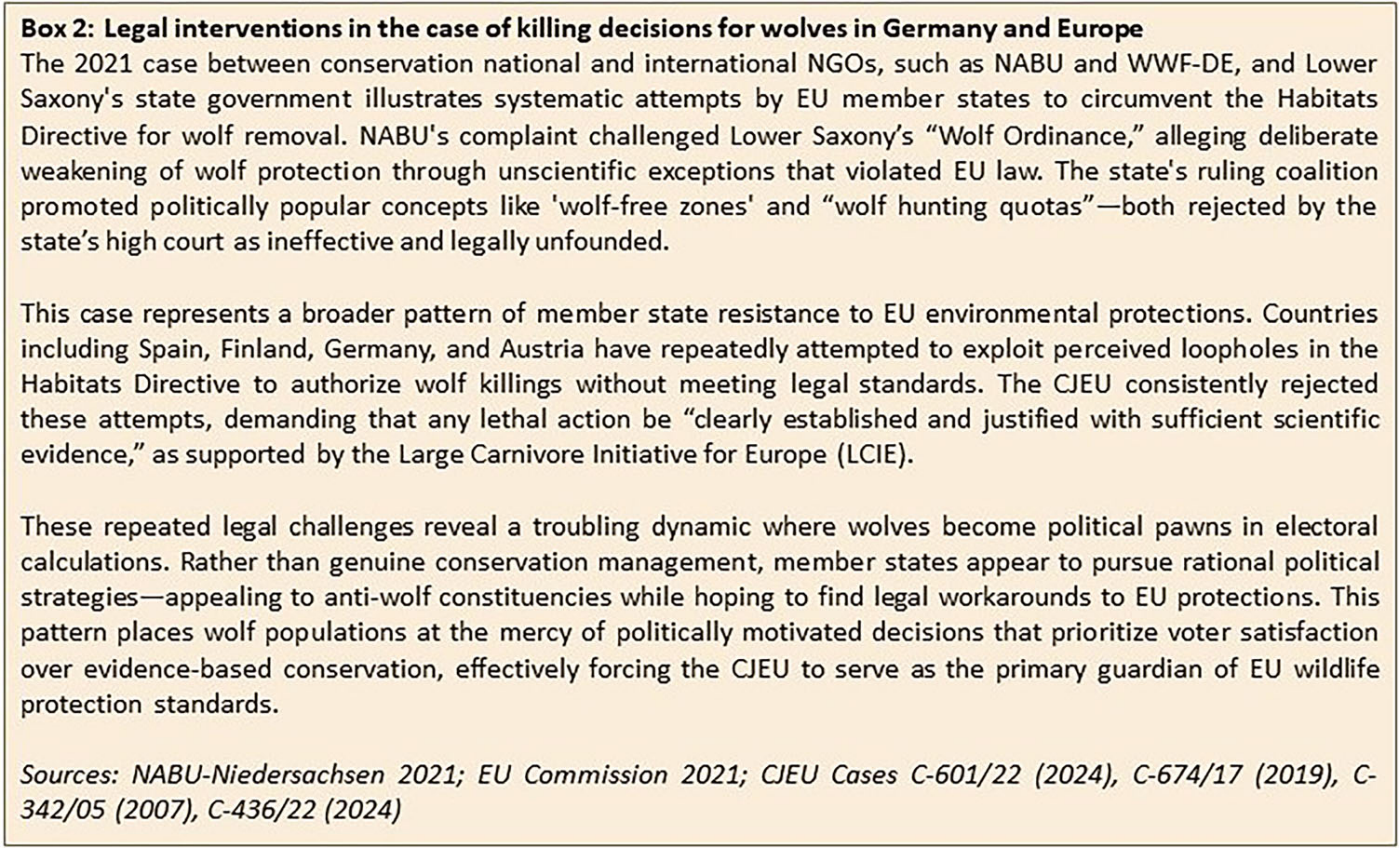


#### Societal responses and conflict escalation

Documentary evidence and interview responses reveal distinct societal response patterns. In India, post-implementation responses typically involve localized protests or support depending on community tolerance levels. Media coverage of the T1 case showed polarized reactions, with wildlife activists filing contempt proceedings while affected communities supported the action. One researcher [IN_010] observed: “*After tiger removals, communities usually calm down, but activists become more vocal*.” Also refer to Box 1 with details on the case of tigress T1 that reflects on the implementation challenges and how Indian societies react to the situation.

German cases demonstrate sustained polarization regardless of implementation outcomes. Court documents show conservation groups consistently challenge removals through administrative and civil courts, while agricultural representatives lobby for expanded management authority. Media analysis by interviewees [DE_021] indicated that “*each wolf incident generates weeks of polarized coverage, regardless of whether wolves are actually removed*.” This pattern suggests that societal responses are driven more by ideological positions than by management outcomes themselves.

## Discussion

### Contextual determinants of lethal control frameworks

The comparative analysis of this study reveals that lethal control decisions in human–carnivore conflict management are fundamentally shaped by the interaction of ecological, sociocultural and institutional–political contexts rather than by standardized biological or conservation criteria alone. The contrasting approaches between India's tiger management and Germany's wolf management demonstrate how similar conservation challenges produce divergent policy outcomes when embedded within different contextual frameworks.

The ecological–physical context establishes the foundational parameters for human–carnivore interactions, with both countries experiencing conflicts in human-modified landscapes characterized by habitat fragmentation and high population densities (Warrier et al. 2020; Planillo et al. 2024). However, the historical continuity of carnivore presence emerges as one of the critical differentiating factors. India's uninterrupted tiger presence, despite dramatic population fluctuations, has maintained institutional memory and adaptive capacity within both communities and governance structures (Jhala et al. 2025). Conversely, Germany's two-century absence of wolves created an institutional and social knowledge vacuum that continues to complicate management responses, supporting previous findings that historical wildlife relationships significantly influence contemporary conservation outcomes (Chapron and Lopez-Bao 2014). This can be illustrated by Pettersson et al. (2022), who examined human–wolf coexistence in Spain and found significant differences in sociocultural attitudes between regions with and without wolf populations. In north-western Spain, where wolves have maintained a continuous presence, communities have preserved traditional ecological knowledge and livestock protection practices, enabling them to manage risk and accept wolves as part of the landscape. Conversely, in southern regions where wolves have been absent or are only recently returning, the erosion of traditional knowledge and practical experience has weakened coping mechanisms, resulting in heightened fear, conflict, and resistance among local stakeholders. This is reflected in the institutional experience, where policies aligning to existing traditional practices in the north promotes trust-based relationships, conversely in the south, such policies are received as external solutions, imposed on the stakeholders, thereby losing legitimacy of governance.

The sociocultural context proves equally determinative, with cultural attitudes toward carnivores directly translating into tolerance thresholds and policy support (Struebig et al. 2018). India's complex cultural integration of tigers into religious and mythological narratives creates what we term “embedded tolerance" a baseline acceptance of carnivore presence that requires extraordinary circumstances (multiple human fatalities) to override (Jhala et al. 2025). This cultural embeddedness aligns with broader patterns observed across traditional societies where wildlife holds spiritual significance (Oommen 2021). Germany's historical framing of carnivores as antagonistic entities, combined with contemporary urban–rural polarization, creates "conditional tolerance" that varies significantly across demographic and political lines, consistent with findings on European carnivore attitudes (Arbieu et al. 2019).

### Institutional architectures and decision-making efficacy

The institutional–political context emerges as perhaps the most influential determinant of lethal control implementation, with governance structures, legal frameworks, and political dynamics creating distinct operational environments. India's quasi-federal system with decentralized authority within each state vested in Chief Wildlife Wardens enables rapid decision-making during crisis situations, though implementation effectiveness varies significantly with resource availability and therefore institutional capacity (Rastogi et al. 2014; Das 2019; Srivathsa et al. 2022). This model provides clear accountability chains but vulnerability to localized political pressures remain.

Germany's federal system embedded within EU environmental law creates multiple accountability layers that, while providing robust protection against illegitimate removals, simultaneously generate implementation gridlock (Epstein et al. 2019). The progressive narrowing of permissible justifications through CJEU rulings (and local court interpretations of what these rulings mean) represents a sophisticated attempt to prevent political exploitation of derogation mechanisms, yet paradoxically reduces management flexibility to address legitimate conflicts (Epstein et al. 2015). This tension between legal protection and practical management reflects broader challenges in multi-level governance systems where local needs must conform to supranational frameworks (Trouwborst and Fleurke 2019).

The recent down-listing of wolves from “strictly protected” to “protected” status under the Bern Convention and EU Habitats Directive illustrates the dynamic nature of institutional frameworks and their political responsiveness. However, the maintenance of conservation objectives and the requirement for favorable conservation status suggests that fundamental protective mechanisms remain intact, potentially limiting practical management changes (as presented from the Case C-436/22 (CJEU 2024).

### Evidence standards and implementation challenges

Our findings reveal both commonalities and case-specific disparities in evidentiary requirements and verification processes that directly impact implementation effectiveness. Both India and Germany rely on technologically intensive approach for field-based evidence collection, coupled with genetic analysis. While pragmatic, temporal delays can exacerbate conflict situations. In both cases, challenges arise with the implementation due to field conditions (such as in the case of *Avni*—despite military-level operations, the tigress could not be captured, while in Germany, the inability to readily identify responsible individuals despite genetic confirmation of species involvement creates a fundamental implementation bottleneck). However, in India operational feasibility depends heavily on personnel training and institutional capacity (Srivathsa et al. 2022). Whereas in Germany, bureaucratic process limits timely response to management implementation (Köck 2019).

Together with the institutional architectures of decision-making process the approaches to management standards reflect different prioritizations within the evidence-policy interface: India prioritizes rapid response capability to protect human life, while Germany emphasizes forensic certainty to prevent unjustified removals. Neither approach fully resolves the tension between scientific rigor and management practicality, suggesting the need for adaptive frameworks that can accommodate contextual constraints while maintaining evidence-based decision-making (Das 2019; Epstein et al. 2019).

### Political dynamics and legitimacy construction

The politicization of carnivore management emerges as a critical factor distinguishing the two cases, with implications for policy stability and implementation success. India's tendency toward localized, reactive politicization around specific incidents allows for crisis-driven responses while maintaining broader conservation consensus (Marguiles 2019). Political pressure manifests primarily during acute conflict episodes, creating temporal windows for management action without fundamentally challenging conservation frameworks. Moreover, cultural acceptance to authority, and psychological coping mechanisms, despite hardships arising from conservation policies, gives legitimacy to the department and their administration (Martin et al. 2018).

Germany's systematic politicization of wolf management along ideological and demographic lines transforms individual conflict incidents into proxy battles for broader sociopolitical tensions. This pattern, where carnivore management becomes symbolic of rural–urban divisions and environmental governance philosophy, creates persistent implementation challenges regardless of specific management outcomes. The result is policy paralysis where rational management decisions become impossible due to polarized stakeholder positions (Märcz and Gibbert 2023).

The role of media in shaping public perception and political discourse varies significantly between contexts. India's media coverage tends to be incident-specific and reactive, while German media coverage reflects and reinforces broader ideological polarization. This difference suggests that media influence on carnivore management is not uniform but depends on underlying social and political structures.

### Adaptive management and policy learning

Our analysis reveals limited evidence of systematic policy learning and adaptive management in either context, despite ongoing implementation challenges. Both countries demonstrate primarily reactive approaches to conflict management, with preventive measures and social dimensions of conservation receiving insufficient attention relative to crisis response mechanisms. This pattern suggests that institutional incentives favor visible response to dramatic incidents over less visible but potentially more effective prevention strategies.

The persistence of implementation challenges—evidence collection delays in India and bureaucratic gridlock in Germany—indicates that learning from operational experience is not effectively translating into policy refinement. This may reflect broader institutional inertia or the dominance of crisis-driven decision-making that prioritizes immediate responses over systematic improvement.

However, emerging recognition of these challenges, as evidenced in stakeholder interviews, suggests potential pathways for improvement. Indian experts' emphasis on landscape-level planning and technological early-warning systems, combined with German experts' focus on public education and coexistence strategies, indicate awareness of current limitations and possible solutions.

### Building sustainable coexistence and policy implications

Our comparative analysis reveals critical insights into the conditions that foster or undermine sustainable coexistence between humans and large carnivores. The contrasting outcomes between India and Germany demonstrate that coexistence is not merely a conservation ideal but a dynamic socio-ecological process requiring continuous negotiation and adaptation across multiple scales.

India's embedded tolerance, rooted in cultural integration and historical continuity, represents a form of coexistence that has demonstrated remarkable persistence despite escalating conflict pressures. However, this system shows signs of strain as traditional tolerance thresholds are exceeded, culturally robust coexistence frameworks require active management and adaptation to maintain stability (Macura et al. 2016). The reactive nature of India's approach—intervening only after tolerance limits are breached—indicates that coexistence systems require proactive mechanisms to prevent threshold exceedance rather than simply responding to system failure (Menon and Borah 2024). This can be achieved at socio-economical (Das 2019; Rai et al. 2021), ecological (Jhala et al 2021), and institutional grounds (Srivastava et al. 2025). The current framing of tiger conservation policies in India, guided by strong political will (Carbone et al. 2019; Jhala et al. 2021) and low agency for rural communities (Menon and Borah 2024) needs a shift for social justice (Mathur 2019; Deshpande 2023). This requires reforms in top-down state administered conflict mitigation strategies to inclusive and active participatory frameworks that allow the department and local communities to work together, where powers in policy decisions are balanced (Kashwan 2016; Srivastava et al. 2025).

Germany's experience illustrates how the absence of historical coexistence relationships creates fragile foundations for contemporary coexistence efforts. The polarized stakeholder positions and implementation gridlock suggest that newly established coexistence systems are particularly vulnerable to external shocks and require deliberate institution building to develop adaptive capacity (König et al. 2023). The emphasis on technological solutions (genetic verification, preventive measures) without corresponding investment in social acceptance mechanisms reveals the limitations of purely technical approaches driven by political motifs to coexistence challenges. To promote coexistence with wolves in Germany, active measures are needed in creating positive narratives for wolves (Jürgens et al. 2023). This can be achieved by sensitizing and educating media on wolf ecology, benefits of installing preventive measures; involving people in conservation through citizen science projects (Ostermann-Miyashita et al. 2025).

Both cases demonstrate that sustainable coexistence requires institutional frameworks capable of managing the inherent tension between conservation objectives and human interests while maintaining legitimacy across diverse stakeholder groups. The key appears to lie in developing adaptive governance mechanisms that can respond to changing conditions while preserving the core elements that enable human–carnivore coexistence to persist over time. This could be achieved by investigating in the traditional knowledge of coexistence in both countries and development of integrated conservation governance frameworks (Sarker et al. 2025). In case of Germany, examples of traditional knowledge of human–wolf coexistence from EU member states (ex., Pettersson et al. 2022; Pérez-Barbería and Bodas 2025) and/or historical anecdotes of cultural knowledge of “living with wolves” from German literature could help achieve legitimacy to conflict prevention and mitigation strategies in the country. This could ensure a proactive approach to human–carnivore coexistence that serves both conservation goals and community safety effectively.

These insights have significant implications for carnivore conservation policy development, particularly regarding the design of lethal control frameworks. The comparative analysis demonstrates that effective policy design must account for contextual variables rather than assuming universal applicability of management approaches. Successful frameworks appear to require alignment between legal mechanisms, institutional capacity, cultural attitudes and political dynamics. The tension between conservation objectives and conflict management necessitates context-specific equilibrium points that balance carnivore protection with human interests. Neither extreme—absolute protection regardless of circumstances nor unrestricted management authority—appears sustainable given the complex sociopolitical environments in which carnivore conservation operates.

## Conclusion

This comparative study reveals that lethal control decision-making in human–carnivore conflict management is fundamentally shaped by the complex interplay of ecological, sociocultural and institutional–political contexts rather than by standardized conservation criteria. The contrasting approaches between India's tiger management and Germany's wolf management demonstrate how similar conservation challenges produce divergent policy outcomes when embedded within different contextual frameworks.

Our application of the Institutional Analysis and Development framework successfully illuminated the mechanisms through which contextual variables influence decision-making processes and implementation outcomes. The analysis reveals that effective carnivore conservation policy requires careful alignment between legal frameworks, institutional capacity, cultural attitudes and political dynamics rather than assuming universal applicability of management approaches.

The study identifies several key factors that determine implementation success: historical continuity of carnivore presence influences institutional memory and adaptive capacity; cultural attitudes toward carnivores directly translate into tolerance thresholds and policy support; governance structures determine decision-making efficiency and accountability; evidentiary requirements create trade-offs between scientific rigor and operational feasibility; and political dynamics can either enable crisis-driven responses or create systematic implementation gridlock.

These findings have important implications for conservation policy development globally. As human–carnivore conflicts intensify with growing carnivore populations and expanding human development, understanding the contextual determinants of management decisions becomes critical for developing effective and sustainable conservation strategies. Future research should focus on developing adaptive frameworks that can accommodate contextual constraints while maintaining evidence-based decision-making and conservation effectiveness.

The persistence of implementation challenges in both countries highlights the need for systematic policy learning and adaptive management approaches that can evolve based on operational experience. This requires institutional mechanisms that prioritize prevention over crisis response and facilitate evidence-based policy refinement over time. Our results suggest that future policy development should focus on building institutional capacity for preventive management, improving evidence-based decision-making processes, and developing stakeholder engagement mechanisms that can accommodate diverse perspectives while maintaining conservation effectiveness.

Ultimately, our study demonstrates that the binary debate of "kill versus don't kill" oversimplifies the complex reality of carnivore management decisions. Instead, effective conservation requires nuanced approaches that acknowledge contextual constraints while working toward coexistence frameworks that balance conservation objectives with legitimate human concerns. Such approaches demand continued investment in institutional capacity, stakeholder engagement and evidence-based policy development tailored to specific socio-ecological contexts. Further research could explore how these institutional dynamics apply to other species globally.

## Supplementary Information

Below is the link to the electronic supplementary material.

Supplementary file1 (PDF 1205 KB)

## References

[CR1] Ahmed, T., and A. Khan. 2022. Economics of carnivore depredation: A case study from the northern periphery of Corbett Tiger Reserve, Uttarakhand, India. *Acta Ecologica Sinica* 42: 68–73. 10.1016/j.chnaes.2021.03.007.

[CR2] Aiyadurai, A. 2021. The real Sherni: How Avni put the spotlight on the complex nature of tiger conservation in India. Scroll.in. Available at: https://scroll.in/article/999244/the-real-sherni-how-avni-put-the-spotlight-on-the-complex-nature-of-tiger-conservation-in-india, Accessed 27 May 2025; 17: 12 CET.

[CR4] Arbieu, U., M. Mehring, N. Bunnefeld, P. Kaczensky, I. Reinhardt, H. Ansorge, K. Böhning-Gaese, J. A. Glikman, et al. 2019. Attitudes towards returning wolves (Canis lupus) in Germany: Exposure, information sources and trust matter. *Biological Conservation* 234: 202–210. 10.1016/j.biocon.2019.03.027.

[CR5] Arora, B., and K. K. Kailash. 2018. Beyond quasi federalism: Change and continuity in Indian federalism. *Studies in Indian Politics* 6: 297–302. 10.1177/2321023018797560.

[CR6] Bartlett, L., and F. Vavrus. 2016. *Rethinking case study research: A comparative approach*. Routledge. 10.4324/9781315674889.

[CR7] Bartlett, L., and F. Vavrus. 2017. Comparative case studies: An innovative approach. *Nordic Journal of Comparative and International Education (NJCIE)* 1. 10.7577/njcie.1929.

[CR8] Beggiora, S., and V. M. Exley. 2024. Coexistence in the Anthropocene: Tigers and Humans of the Dibang Valley. *The Oriental Anthropologist* 24: 0972558X241227858. 10.1177/0972558X241227858.

[CR9] Bhardwaj, M. 2021. Human-Tiger Conflicts Continues in Kodagu With 3 Human Kills, Green Minute, available from https://greenminute.in/2021/03/09/human-tiger-conflict-continues-in-kodagu-with-3-human-kills/. Accessed 21 May 2024; 16: 44 CET.

[CR10] Bilalova, S., J. Newig, and S. Villamayor-Tomas. 2025. Toward Sustainable Water Governance? Taking Stock of Paradigms, Practices, and Sustainability Outcomes. *Wiley Interdisciplinary Reviews: Water* 12: e1762. 10.1002/wat2.1762.

[CR11] Boitani, L., and J. D. Linnell. 2015. Bringing large mammals back: Large carnivores in Europe. *Rewilding European Landscapes*, pp. 67–84. 10.1007/978-3-319-12039-3.

[CR12] Bombieri, G., V. Penteriani, K. Almasieh, H. Ambarlı, M. R. Ashrafzadeh, C. S. Das, N. Dharaiya, R. Hoogesteijn, et al. 2023. A worldwide perspective on large carnivore attacks on humans. *PLoS Biology* 21: e3001946. 10.1371/journal.pbio.3001946. 36719873 10.1371/journal.pbio.3001946 PMC9888692

[CR13] Brandenburg - Wolf Management Plan. 2019. Available from https://www.dbb-wolf.de/wolf-management/wolf-management-of-federal-states/management-plans. Accessed 30 Jan 2025; 16:52 CET.

[CR14] Braun, V., and V. Clarke. 2006. Using thematic analysis in psychology. *Qualitative Research in Psychology* 3: 77–101. 10.1191/1478088706qp063oa.

[CR100] Carbone, C., M. Hayward, and J. Bump. 2019. India keeps a close eye on its tigers. *Nature* 572: 586–587. 10.1038/d41586-019-02546-z. 10.1038/d41586-019-02546-z 31455912

[CR15] Carter, N. H., and J. D. Linnell. 2016. Co-adaptation is key to coexisting with large carnivores. *Trends in Ecology & Evolution* 31: 575–578. 10.1016/j.tree.2016.05.006. 27377600 10.1016/j.tree.2016.05.006

[CR16] Carter, N. H., and J. D. Linnell. 2023. Building a resilient coexistence with wildlife in a more crowded world. *PNAS Nexus* 2: pgad030. 10.1093/pnasnexus/pgad030. 36896129 10.1093/pnasnexus/pgad030 PMC9991453

[CR17] Chapron, G., and J. V. López-Bao. 2014. Conserving carnivores: Politics in play. *Science* 343: 1199–1200. 10.1126/science.343.6176.1199-b. 10.1126/science.343.6176.1199-b 24626913

[CR18] Chatterjee, S. 2023. Rising trend of man-tiger conflict at man-nature interface of Indian Sundarbans: Study towards traditional understanding and challenging livelihood of Sundarbans people. *Safety in Extreme Environments* 5: 35–46. 10.1007/s42797-023-00069-5.

[CR19] Court of Justice of the European Union (CJEU). 2024. ASCEL versus Administración de la Comunidad Autónoma de Castilla y León. Case number - C-436/22. Available at: https://eur-lex.europa.eu/legal-content/EN/TXT/?uri=CELEX%3A62022CC0436&qid=1748365576642. Accessed 29 May 2025; 14:00 CET.

[CR20] Council of Europe. 2025. Modification of wolf protection under the Bern Convention enters into force. Available at: https://www.coe.int/en/web/portal/-/modification-of-wolf-protection-under-the-bern-convention-enters-into-force (last assessed on 19.05.2025; 08:10 CET).

[CR21] Cusack, J. J., E. B. Nilsen, M. F. Israelsen, H. Andrén, M. Grainger, J. D. Linnell, J. Odden, and N. Bunnefeld. 2022. Quantifying the checks and balances of collaborative governance systems for adaptive carnivore management. *Journal of Applied Ecology* 59: 1038–1049. 10.1111/1365-2664.14113. 35910004 10.1111/1365-2664.14113 PMC9306889

[CR22] Das, B. K. 2019. Denial of rights continues: How legislation for ‘democratic decentralisation’ of forest governance was subverted in the implementation process of the forest rights act in India. *The European Journal of Development Research* 31: 957–983. 10.1057/s41287-019-0195-2.

[CR23] DBBW. 2025. Wolf occurrence in Germany. Available at: https://www.dbb-wolf.de/wolf-occurrence. Accessed 07 Feb 2025; 15: 30 CET.

[CR24] de Boon, A., C. Sandström, U. Arbieu, I. Hansen, L. Lehnen, A. Marino, M. Pohja-Mykrä, C. Risvoll, et al. 2021. Governing dual objectives within single policy mixes: An empirical analysis of large carnivore policies in six European countries. *Journal of Environmental Policy & Planning* 23: 399–413. 10.1080/1523908X.2020.1841614.

[CR25] Deshpande, H. 2023. Tiger-Human Conflict in Maharashtra: The Controversial Killings of Avni and Other Man-Eaters. Outlook, available from https://www.outlookindia.com/national/the-tiger-human-conflict-in-maharashtra-the-controversial-killing-of-avni-and-other-man-eaters-news-273673. Accessed 21 May 2024; 16:41 CET.

[CR26] Di Bernardi, C., G. Chapron, P. Kaczensky, F. Álvares, H. Andrén, V. Balys, J. C. Blanco, S. Chiriac, et al. 2025. Continuing recovery of wolves in Europe. *PLOS Sustainability and Transformation* 4: e0000158. 10.1371/journal.pstr.0000158.

[CR27] Epstein, Y. 2017. Killing wolves to save them? Legal responses to ‘tolerance hunting’ in the European Union and United States. *Review of European, Comparative & International Environmental Law* 26: 19–29. 10.1111/reel.12188.

[CR28] Epstein, Y., J. V. López-Bao, and G. Chapron. 2015. A legal-ecological understanding of favorable conservation status for species in Europe. *Conservation Letters* 9: 81–88. 10.1111/conl.12200.

[CR29] Epstein, Y., J. V. López-Bao, A. Trouwborst, and G. Chapron. 2019. EU Court: Science must justify future hunting. *Science* 366: 961–961. 10.1126/science.aaz8424. 10.1126/science.aaz8424 31753989

[CR30] European Commission. 2025. Proposal for a DIRECTIVE OF THE EUROPEAN PARLIAMENT AND OF THE COUNCIL amending Council Directive 92/43/EEC as regards the protection status of the wolf (Canis lupus). File number: 2025/0058 (COD). Available at: https://www.europarl.europa.eu/RegData/docs_autres_institutions/commission_europeenne/com/2025/0106/COM_COM(2025)0106_EN.pdf. Accessed 28 May 2025; 16:35 CET.

[CR31] European Parliament and the Council. 2025. Directive (EU) 2025/1237 of 17 June 2025 amending Council Directive 92/43/EEC as regards the protection status of the wolf (*Canis lupus*). *Official Journal of the European Union*, L, 2025/1237. Available at: https://eur-lex.europa.eu/legal-content/EN/TXT/HTML/?uri=OJ:L_202501237. Accessed 13 Oct 2025, 13:00 CET.

[CR32] Federal Nature Conservation Act. 2009. Act on Nature Conservation and Landscape Management (BNatSchG) of 29 July 2009. Available at: https://www.bmuv.de/fileadmin/Daten_BMU/Download_PDF/Naturschutz/bnatschg_en_bf.pdf. Accessed 12 Dec 2024; 14:00 CET.

[CR33] Guillot, L. 2025. Groups take legal action to protect wolves from looser hunting rules. Politico. Available at: https://www.politico.eu/article/ngos-to-take-legal-action-to-protect-wolves-from-looser-hunting-rules/. Accessed 27 May 2025; 18:11 CET.

[CR34] Jarausch, A., V. Harms, G. Kluth, I. Reinhardt, and C. Nowak. 2021. How the west was won: Genetic reconstruction of rapid wolf recolonization into Germany’s anthropogenic landscapes. *Heredity* 127: 92–106. 10.1038/s41437-021-00429-6. 33846578 10.1038/s41437-021-00429-6 PMC8249462

[CR35] Jeffery, C., N. M. Pamphilis, C. Rowe, and E. Turner. 2014. Regional policy variation in Germany: The diversity of living conditions in a ‘unitary federal state.’ *Journal of European Public Policy* 21: 1350–1366. 10.1080/13501763.2014.923022.

[CR36] Jhala, Y., R. Gopal, V. Mathur, P. Ghosh, H. S. Negi, S. Narain, S. P. Yadav, A. Malik, et al. 2021. Recovery of tigers in India: Critical introspection and potential lessons. *People and Nature* 3: 281–293. 10.1002/pan3.10177.

[CR37] Jhala, Y. V., N. A. Mungi, R. Gopal, and Q. Qureshi. 2025. Tiger recovery amid people and poverty. *Science* 387: 505–510. 10.1126/science.adk4827.39883754

[CR38] Jolly, H., T. Satterfield, M. Kandlikar, and S. Tr. 2022. Indigenous insights on human–wildlife coexistence in southern India. *Conservation Biology* 36: e13981. 10.1111/cobi.13981. 36000317 10.1111/cobi.13981

[CR99] Jürgens, U.M., M. Grinko, A. Szameitat, L. Hieber, R. Fischbach, and M. Hunziker. 2023. Managing wolves is managing narratives: Views of wolves and nature shape people’s proposals for navigating human-wolf Relations. *Human Ecology* 51: 35–57. 10.1007/s10745-022-00366-w.

[CR39] Kaczensky, P., N. Ranc, J. Hatlauf, J. C. Payne, I. Acosta-Pankov, N. Drouet-Hoguet, P. Y. Quenette, J. Sentilles et al. 2024. Large carnivore distribution maps and population updates 2017 – 2022/23. Report to the European Commission under contract N° 09.0201/2023/907799/SER/ENV.D.3 “Support for Coexistence with Large Carnivores”, “B.4 Update of the distribution maps”. IUCN/SSC Large Carnivore Initiative for Europe (LCIE) and Istituto di Ecologia Applicata (IEA). https://hdl.handle.net/11250/3174149.

[CR40] Kaltenborn, B. P., and J. D. Linnell. 2022. The coexistence potential of different wildlife conservation paradigms in a historical perspective. *Frontiers in Conservation Science.* 2: 711480. 10.3389/fcosc.2021.711480.

[CR41] Kashwan, P. 2016. Power asymmetries and institutions: Landscape conservation in central India. *Regional Environmental Change* 16: 97–109. 10.1007/s10113-015-0925-8.

[CR42] Khan, A. 2022. Inside the Heartbreaking and Controversial Hunt for a Tigress Who Allegedly Killed 13 People. Vice. Available at: https://www.vice.com/en/article/hunt-for-maneating-tiger-avni-wildlife-conservation-india/ (last assessed on 19.05.2025; 10:30 CET).

[CR43] Khorozyan, I., and M. Heurich. 2022. Large-scale sheep losses to wolves (Canis lupus) in Germany are related to the expansion of the wolf population but not to increasing wolf numbers. *Frontiers in Ecology and Evolution* 10: 778917. 10.3389/fevo.2022.778917.

[CR44] Kiffner, C., G. Chapron, and H. J. König. 2019. Germany’s wolves in the crosshairs. *Science* 365: 1089–1089. 10.1126/science.aay8053. 10.1126/science.aay8053 31515376

[CR45] Kiffner, C., S. Uthes, E. F. Ostermann-Miyashita, V. Harms, and H. J. König. 2022. Patterns of livestock loss associated with a recolonizing wolf population in Germany. *Frontiers in Conservation Science* 3: 989368. 10.3389/fcosc.2022.989368.

[CR46] Köck, W. 2019. Wolf conservation and removal of wolves in Germany–status quo and prospects. *Journal for European Environmental & Planning Law* 16: 262–278. 10.1163/18760104-01603004.

[CR47] König, H. J., C. Kiffner, S. Kramer-Schadt, C. Fürst, O. Keuling, and A. T. Ford. 2020. Human–wildlife coexistence in a changing world. *Conservation Biology* 34: 786–794. 10.1111/cobi.13513. 32406977 10.1111/cobi.13513

[CR48] König, H. J., C. Kiffner, K. Kuhls, S. Uthes, V. Harms, and R. Wieland. 2023. Planning for wolf-livestock coexistence: Landscape context predicts livestock depredation risk in agricultural landscapes. *Animal* 17: 100719. 10.1016/j.animal.2023.100719. 36801550 10.1016/j.animal.2023.100719

[CR49] Linnell, J. D., E. Kovtun, and I. Rouart. 2021. Wolf attacks on humans: an update for 2002–2020. Norwegian Institute for Nature Research (NINA). https://hdl.handle.net/11250/2729772.

[CR50] Linnell, J., A. Trouwborst, and F. Fleurke. 2017. September. When is it acceptable to kill a strictly protected carnivore? Exploring the legal constraints on wildlife management within Europe's Bern Convention. In Exploring the Legal Constraints on Wildlife Management within Europe's Bern Convention (September 13, 2017) (Vol. 12, No. 21, pp. 129–157). 10.3897/natureconservation.21.12836.

[CR51] Lorand, C., A. Robert, A. Gastineau, J. B. Mihoub, and C. Bessa-Gomes. 2022. Effectiveness of interventions for managing human-large carnivore conflicts worldwide: Scare them off, don’t remove them. *Science of the Total Environment* 838: 156195. 10.1016/j.scitotenv.2022.156195. 35623521 10.1016/j.scitotenv.2022.156195

[CR52] Lute, M. L., N. H. Carter, J. V. López-Bao, and J. D. Linnell. 2018. Conservation professionals agree on challenges to coexisting with large carnivores but not on solutions. *Biological Conservation* 218: 223–232. 10.1016/j.biocon.2017.12.035.

[CR53] Lower Saxony Wolf Management plan. 2022. Available from https://www.dbb-wolf.de/wolf-management/wolf-management-of-federal-states/management-plans. Accessed 24 Feb 2025; 16: 55 CET.

[CR54] Macura, B., L. Secco, E. Pisani, A. S. Pullin, and V. Reyes-García. 2016. All that glitters is not gold: The effect of top-down participation on conservation knowledge, attitudes and institutional trust in a Central Indian tiger reserve. *Regional Environmental Change* 16: 125–140. 10.1007/s10113-016-0978-3.

[CR55] Margulies, J. D. 2019. Making the ‘man-eater’: Tiger conservation as necropolitics. *Political Geography* 69: 150–161. 10.1016/j.polgeo.2018.12.011.

[CR56] Martin, A., R. Myers, and N. M. Dawson. 2018. The park is ruining our livelihoods. We support the park! Unravelling the paradox of attitudes to protected areas. *Human Ecology* 46: 93–105. 10.1007/s10745-017-9941-2. 10.1007/s10745-017-9941-2 PMC5849646 29576674

[CR57] Mathur, N. 2019. A Petition to Kill: Efficacious arzees against big cats in India. *Modern Asian Studies* 53: 278–311. 10.1017/S0026749X18000124.

[CR58] Märcz, L., and M. Gibbert. 2023. Fear of the Wolf: Are Human-Wildlife Conflicts Actually Human-Human Feuds? *Society & Animals* 32: 786–805.

[CR59] Mech, L. D. 2017. Where can wolves live and how can we live with them? *Biological Conservation* 210: 310–317. 10.1016/j.biocon.2017.04.029.

[CR60] Menon, A., and R. Borah. 2024. Tiger conservation, biopolitics and the future of Indian environmentalism. *Singapore Journal of Tropical Geography* 45: 70–84. 10.1111/sjtg.12525.

[CR61] Mishra, S. 2023. Don’t kill but catch big cat: HC after shoot-at-sight order. The Times of India, Dec 15, 2023. Available at: https://timesofindia.indiatimes.com/city/dehradun/dont-kill-but-catch-big-cat-hc-after-shoot-at-sight-order/articleshow/106004889.cms?utm_source=chatgpt.com [last assessed om 09.10.2025; 17:00 CET].

[CR62] NABU Niedersachsen / WWF Deutschland / Freundeskreis freilebender Wölfe e. V. 2021. *Normenkontrollklage gegen die Wolfsverordnung Niedersachsen beim OVG Lüneburg eingereicht*. Higher Administrative Court of Lower Saxony press release or official announcement [online]. Available at: https://niedersachsen.nabu.de/tiere-und-pflanzen/saeugetiere/wolf/schutzstatus/31343.html. Accessed 25 July 2025; 14 CET.

[CR63] National Tiger Conservation Authority. 2007. Declaring big cats as man-eaters. F.No. 15–13/2007-NTCA. Available from https://ntca.gov.in/assets/uploads/sops/Guidelines_bigger_cats_man.pdf. Accessed 22 May 2024; 16:10 CET.

[CR64] National Tiger Conservation Authority. 2013. Standard operating procedure to deal with emergency arising due to straying of tigers in human dominated landscapes. F. No. 15–37/2012-NTCA. Available from https://ntca.gov.in/assets/uploads/sops/SOp_Straying_Tiger.pdf. Accessed 06 Dec 2024; 16:04 CET.

[CR65] Niedziałkowski, K. 2022. Between Europeanisation and politicisation: Wolf policy and politics in Germany. *Environmental Politics* 32: 793–814. 10.1080/09644016.2022.2127646.

[CR66] Nyaupane, G. P., S. Poudel, and A. York. 2022. Governance of protected areas: An institutional analysis of conservation, community livelihood, and tourism outcomes. *Journal of Sustainable Tourism* 30: 2686–2705. 10.1080/09669582.2020.1858089.

[CR67] Oommen, M. A. 2021. Beasts in the garden: Human-wildlife coexistence in India’s past and present. *Frontiers in Conservation Science* 2: 703432. 10.3389/fcosc.2021.703432.

[CR68] Ostermann-Miyashita, E. F., H. Kirkland, A. Eklund, D. Hare, H. A. Jansman, C. Kiffner, J. D. Linnell, R. Rigg, et al. 2025. Bridging the gap between science, policy and stakeholders: Towards sustainable wolf–livestock coexistence in human-dominated landscapes. *People and Nature*. 10.1002/pan3.10786.

[CR69] Ostrom, E., R. Gardner, and J. Walker. 1994. *Rules, games, and common-pool resources*. University of Michigan press.

[CR70] Ostrom, E. 2005. Doing Institutional Analysis: Digging Deeper than Markets and Hierarchies, 819 ff. *Menard/shirley*. 10.1007/978-3-031-50810-3_5.

[CR71] Panwar, H. S. 1982. What to do when you've succeeded: Project Tiger ten years later. *Ambio*, pp. 330–337. https://www.jstor.org/stable/4312836.

[CR72] Pates, R., and J. Leser. 2021. *The wolves are coming back: The politics of fear in Eastern Germany*. Manchester University Press.

[CR73] Pérez-Barbería, F. J., and R. Bodas. 2025. Environmental and Societal Impacts of Protecting Traditional Pastoralism from Wolf Predation in Spain. *Sustainability* 17: 8189. 10.3390/su17188189.

[CR74] Pettersson, H. L., C. H. Quinn, G. Holmes, and S. M. Sait. 2022. “They belong here”: Understanding the conditions of human-wolf coexistence in North-Western Spain. *Conservation and Society* 20: 113–123. 10.4103/cs.cs_13_21.

[CR75] Planillo, A., M. Wenzler-Meya, I. Reinhardt, G. Kluth, F. U. Michler, N. Stier, J. Louvrier, K. Steyer, et al. 2024. Understanding habitat selection of range-expanding populations of large carnivores: 20 years of grey wolves (Canis lupus) recolonizing Germany. *Diversity and Distributions* 30: 71–86. 10.1111/ddi.13789.

[CR76] Qureshi, Q., Y. V. Jhala, S. P. Yadav, and A. Mallick. 2023. Status of Tigers in India-2022: Photo-captured Tigers, Summary Report. National Tiger Conservation Authority and Wildlife Institute of India, Dehradun (No. 2023/03, p. 5). TR.

[CR77] Rai, N. D., M. S. Devy, T. Ganesh, R. Ganesan, S. R. Setty, A. J. Hiremath, S. Khaling, and P. D. Rajan. 2021. Beyond fortress conservation: The long-term integration of natural and social science research for an inclusive conservation practice in India. *Biological Conservation* 254: 108888. 10.1016/j.biocon.2020.108888.

[CR78] Rastogi, A., G. M. Hickey, R. Badola, and S. A. Hussain. 2014. Understanding the local socio-political processes affecting conservation management outcomes in Corbett Tiger Reserve, India. *Environmental Management* 53: 913–929. 10.1007/s00267-014-0248-4. 24522894 10.1007/s00267-014-0248-4

[CR79] Redpath, S. M., J. D. Linnell, M. Festa-Bianchet, L. Boitani, N. Bunnefeld, A. Dickman, R. J. Gutiérrez, R. J. Irvine, et al. 2017. Don’t forget to look down–collaborative approaches to predator conservation. *Biological Reviews* 92: 2157–2163. 10.1111/brv.12326. 28338282 10.1111/brv.12326

[CR80] Reinhardt, I., G. Kluth, S. Nowak, and R. W. Myslajek, 2013. A review of wolf management in Poland and Germany with recommendations for future transboundary collaboration. Deutschland/Bundesamt für Naturschutz.

[CR81] Rose, L. 2021. *‘Avni’ in Leopard Moon Rising: Distant Views of India*, 25–30. Gritstone Publishing.

[CR82] Rother, E. T. 2007. Systematic literature review X narrative review. *Acta Paulista De Enfermagem* 20: v–vi. 10.1590/S0103-21002007000200001.

[CR83] Sarker, S. K., M. M. Ahsan, A. M. Rashid, A. N. M. Hossain, M. B. Al Mamun, and H. T. Rahman. 2025. Tiger Conservation Governance in the Bangladesh Sundarbans Identifying Inter-Institutional Gaps. In *Institutional Diversity and Sustainable Environmental Management* (pp. 55–72). CRC Press. 10.1201/9781003042914-4.

[CR84] Shukla, P. N., E. C. Rao, and V. K. Mishra. 2025. Factors influencing livestock grazing in Pench Tiger Reserve. *Maharashtra. Discover Environment* 3: 33. 10.1007/s44274-025-00216-8.

[CR85] Soman, D., and V. Anitha. 2020. Community dependence on the natural resources of Parambikulam Tiger Reserve, Kerala, India. *Trees, Forests and People* 2: 100014. 10.1016/j.tfp.2020.100014.

[CR86] Srivastava, N., J. D. C. Linnell, C. Sattler, J. Kleemann, H. J. König, R. Krishnamurthy, and C. Fürst. 2025. Claws, Laws, and Coexistence: Governance of carnivore conservation – the cases of tiger in India and wolf in Germany. Current Research in Environmental Sustainability [submitted].

[CR87] Srivathsa, A., A. Banerjee, S. Banerjee, M. M. Chawla, A. Das, D. Ganguly, R. G. Rodrigues, T. Adhya, et al. 2022. Chasms in charismatic species research: Seventy years of carnivore science and its implications for conservation and policy in India. *Biological Conservation* 273: 109694. 10.1016/j.biocon.2022.109694.

[CR88] Stohr, C., and E. Coimbra. 2013. The governance of the wolf-human relationship in Europe. *Rev. Eur. Stud.* 5: 1. 10.5539/res.v5n4pl.

[CR89] Struebig, M. J., M. Linkie, N. J. Deere, D. J. Martyr, B. Millyanawati, S. C. Faulkner, S. C. Le Comber, F. M. Mangunjaya, et al. 2018. Addressing human-tiger conflict using socio-ecological information on tolerance and risk. *Nature Communications* 9: 1–9. 10.1038/s41467-018-05983-y. 10.1038/s41467-018-05983-y PMC6110717 30150649

[CR90] Trouwborst, A., L. Boitani, and J. D. Linnell. 2017. Interpreting ‘favourable conservation status’ for large carnivores in Europe: How many are needed and how many are wanted? *Biodiversity and Conservation* 26: 37–61. 10.1007/s10531-016-1238-z.

[CR91] Trouwborst, A., and F. M. Fleurke. 2019. Killing wolves legally: Exploring the scope for lethal wolf management under European nature conservation law. *Journal of International Wildlife Law & Policy* 22: 231–273. 10.1080/13880292.2019.1686223.

[CR92] Software, V. E. R. B. I. 2021. *MaxQDA 2022, computer program*. Berlin: VERBI Software.

[CR93] Warrier, R., B. R. Noon, and L. Bailey. 2020. Agricultural lands offer seasonal habitats to tigers in a human-dominated and fragmented landscape in India. *Ecosphere* 11: e03080. 10.1002/ecs2.3080.

[CR94] The Wildlife (Protection) Act. 1972. and subsequent amendments: Wildlife Protection (Amendment) Acts of 2002, 2006, 2022. New Delhi: Government of India. Available at: https://www.indiacode.nic.in/bitstream/123456789/1726/1/a1972-53.pdf. Accessed 21 Nov 2024.

[CR95] World Population Review. 2024. https://worldpopulationreview.com/country-rankings/countries-by-density. Accessed 24 May 2024; 13:10 CET.

[CR96] Worldometer. 2025. https://www.worldometers.info/world-population/. Accessed 24 May 2025; 12:00 CET.

[CR97] Zscheischler, J., M. Busse, and N. Heitepriem. 2019. Challenges to build up a collaborative landscape management (CLM)—lessons from a stakeholder analysis in Germany. *Environmental Management* 64: 580–592. 10.1007/s00267-019-01205-3. 31555874 10.1007/s00267-019-01205-3 PMC6838031

[CR98] Zscheischler, J., and J. Friedrich. 2022. The wolf (canis lupus) as a symbol of an urban–rural divide? Results from a media discourse analysis on the human–wolf conflict in Germany. *Environmental Management* 70: 1051–1065. 10.1007/s00267-022-01719-3. 36155838 10.1007/s00267-022-01719-3 PMC9622530

