# Supplementary material for: Beyond kill or no-kill: Institutional analysis of lethal control decision-making in large carnivore management
Source: Ambio. 2025 Dec 11;55(5):1039–60. doi: 10.1007/s13280-025-02317-3 (PMC13035986; doi:10.1007/s13280-025-02317-3)
Supplement: Supplementary file 1 — Supplementary file1 (PDF 1205 KB) [file 13280_2025_2317_MOESM1_ESM.pdf]

Ambio

Supplementary Information

This supplementary information has not been peer reviewed

Title: Beyond Kill or No-Kill: Institutional Analysis of Lethal Control Decision-Making in Large Carnivore Management

S1: Interview guidelines: Institutional Strategies for Managing Conflict Situations Involving Lethal Control of Problem Carnivores

Study Overview

The interview explores institutional strategies and governance frameworks for managing human-carnivore conflicts, including decision-making processes and institutional arrangements surrounding lethal control of problem carnivore individuals. The study aims to understand how different actors navigate complex governance challenges in situations where lethal control measures are considered or implemented.

Interview Structure

The interview followed exploratory open-ended questionnaire format. Questions targeted on overall governance structure for large carnivore conservation (tiger in India and wolf in Germany), specifically to manage human-carnivore interactions and challenges associated with it. We conducted expert interviews in a "problem-centered expert interview framework" based on Döringer (2021). This allows to structure open-ended interviews by an objective, guided by ad-hoc questions by the interviewer (the first author) to attain implicit knowledge of an expert.

Format: Open-ended, problem-centered expert interview framework
Duration: Approximately 55 minutes
Approach: Five core questions with follow-up probes based on participant responses
Method: Descriptive note-taking (recording only with explicit consent)

Core Interview Questions

1. Institutional Framework for Lethal Control Decisions

Main Question: Can you describe the institutional framework and governance structure that guides decision-making when lethal control of problem carnivores is being considered in your context?

Follow-up Probes:

- Who are the key institutional actors involved in these decisions?
- What are the formal procedures or protocols that must be followed?
- What legal or policy frameworks govern when lethal control can be implemented?

- How do scientific assessments factor into institutional decision-making?

2. Institutional Challenges in Lethal Control Governance

Main Question: What are the primary institutional challenges you have observed in governing situations where lethal control of problem carnivores is considered or implemented?

Follow-up Probes:

- What capacity constraints do institutions face in managing these situations?
- How do political pressures influence institutional decision-making processes?
- What role does public opinion play in institutional responses?

3. Inter-institutional Collaboration and Coordination

Main Question: How do different institutions collaborate when managing conflict situations that may involve lethal control of carnivores?

Follow-up Probes:

- What formal mechanisms exist for inter-institutional coordination?
- How are roles and responsibilities divided among different institutions?
- What information-sharing protocols exist between institutions?
- How do institutions handle jurisdictional overlaps or gaps?
- Can you describe successful examples of institutional collaboration in these contexts?

4. Barriers to Effective Institutional Collaboration

Main Question: What challenges do institutions face in maintaining effective collaboration when dealing with lethal control decisions?

Follow-up Probes:

- What institutional conflicts or tensions arise in these situations?
- What resource or capacity constraints limit collaborative efforts?
- How do legal or regulatory frameworks sometimes hinder collaboration?

5. Institutional Recommendations and Improvements

Main Question: What institutional reforms or strategies would you recommend to better govern conflict situations involving potential lethal control of carnivores?

Follow-up Probes:

- What changes to institutional structures would improve decision-making?
- How could inter-institutional coordination mechanisms be strengthened?
- What new institutional arrangements might be beneficial?
- How could institutions better engage with affected communities and stakeholders?
- What capacity building or training would benefit institutional actors?

Interview Conclusion

Closing Questions:

- Is there anything important about institutional approaches to lethal control governance that we haven't discussed?
- Are there any institutional actors or perspectives we should consider for this study?
- Would you be available for a brief follow-up if we need clarification on any points?

Administrative:

- Confirm anonymization preferences and coding (IN_xxx for India, DE_xxx for Germany)
- Remind about confidentiality protections
- Provide contact information for follow-up questions

Methodological Notes

Sampling Strategy: Purposive snowball sampling targeting institutional actors across:

- Administrators (30%)
- Stakeholder representatives (20%)
- Conservationists (18%)
- Policy-makers (15%)
- Social scientists (11%)
- NGOs (6%)

Data Collection: Descriptive note-taking during interviews, with immediate post-interview transcription and review to ensure completeness.

Ethical Considerations: Written consent obtained, full anonymization provided, adherence to European Guidelines on Data Protection.

Follow-up Strategy: Delphi approach for additional information gathering based on initial analysis findings.

S2: Thematic analysis process and identified themes from qualitative interview data.

The figure illustrates the systematic three-phase analytical approach employed in this study, progressing from transcribed interview data through initial coding and familiarization to comprehensive thematic analysis. Braun and Clarke's (2006) six-phase approach within a qualitative content analysis framework, with themes developed inductively through iterative coding and constant comparison methods.

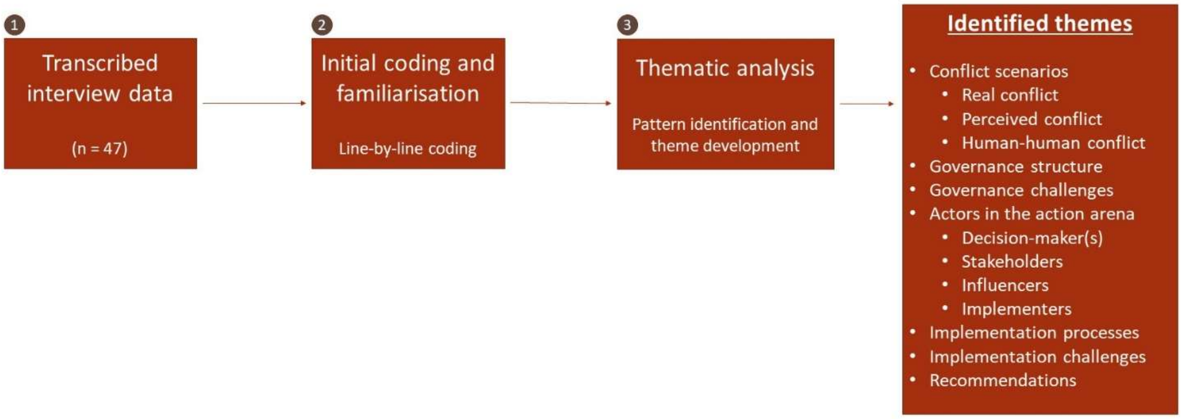

S3: List of documents referred for review of legislative documents related to the management of human-tiger conflicts in India (a) and human-wolf conflicts in Germany (b).

a. India

S. no.	Name of the document	Reference
1.	Wildlife (Protection) Act, 1972	The Wildlife (Protection) Act, 1972, and subsequent amendments: Wildlife Protection (Amendment) Acts of 2002, 2006, 2022. New Delhi: Government of India. Available at: https://www.indiacode.nic.in/bitstream/123456789/1726/1/a1972-53.pdf. [last accessed on 21. 11. 2024].
2.	Wildlife Protection (Amendment) Act, 2002	
3.	Wildlife Protection (Amendment) Act, 2006	
4.	Wildlife Protection (Amendment) Act, 2022	
5.	The Indian Forest Act, 1927	The Indian Forest Act, 1927. Available at: http://nbaindia.org/uploaded/Biodiversityindia/Legal/3.%20Indian%20forest%20act.pdf. [last accessed on 13. 11. 2024].
6.	Scheduled Tribes and other Traditional Forest Dwellers (Forest Rights) Act, 2006	Scheduled Tribes and Other Traditional Forest Dwellers (Forest Rights) Act, 2006. Available at: https://www.indiacode.nic.in/bitstream/123456789/8311/1/a2007-02.pdf. [Last accessed on 21. 11. 2024].

7.	Indian Penal Code, 1860	Indian Penal Code, 1860. Available at: https://www.indiacode.nic.in/bitstream/123456789/4219/1/THE-INDIAN-PENAL-CODE-1860.pdf. [Last accessed on 21. 03. 2024].
8.	Biodiversity (Amendment) Act, 2023	Biodiversity (Amendment) Act, 2023. Available at: https://egazette.gov.in/WriteReadData/2023/247815.pdf. [Last accessed on 22. 03. 2024].
9.	NTCA, 2022 Status of tigers, co-predators and prey	National Tiger Conservation Authority, 2022. Status of Tigers, Co-Predators and Prey in India. Available at: https://ntca.gov.in/assets/uploads/Reports/AITM/status_of_tiger-copredators-2022.pdf. [Last accessed on 13.05. 2024].
10.	National Wildlife Action Plan, 2017-2031	Ministry of Environment, Forest and Climate Change, 2017. National Wildlife Action Plan, 2017-2031. Available at: https://ntca.gov.in/assets/uploads/Reports/Others/Wildlife_Action_Plan_2017_31.pdf [last accessed on 16. 12. 2024; 17:00 CET].
11.	NTCA-MEE-AITE 2024	National Tiger Conservation Authority, 2024. Bridging the gap: Unveiling effectiveness of India's tiger reserve management. Available at: https://ntca.gov.in/assets/uploads/Reports/Others/NTCA_MEE-AITE%20_Compilation_Book-lowres.pdf [last accessed on 18.11.2014; 15:06 CET].
12.	NTCA Guideline Tiger Conservation Plan	National Tiger Conservation Authority, 2007. Guidelines for Preparation of Tiger Conservation Plan. Available at: https://ntca.gov.in/assets/uploads/guidelines/tc_plan.pdf [last accessed on 13.05.2024; 15:30 CET].
13.	National Human-Wildlife Conflict Mitigation Strategy and	Ministry of Environment, Forest and Climate Change, 2021. National Human-Wildlife Conflict Mitigation Strategy and Action Plan of India, 2021-2026. Available at: https://moef.gov.in/uploads/2022/01/National-Human-Wildlife-Conflict-Mitigation-Strategy-and-Action-Plan-of-India-2.pdf [last accessed on 21. 12. 2024; 16:04 CET].

	Action Plan of India, 2021-2026	
14.	NTCA – Guidelines Bigger Cats Man	National Tiger Conservation Authority, 2007. Guidelines for declaring bi cats as maneaters. Available at: https://ntca.gov.in/assets/uploads/sops/Guidelines_bigger_cats_man.pdf [last accessed on 18. 10. 2024; 10:14 CET].
15.	Tiger Conservation Foundation Guidelines, 2007	Ministry of Environment, Forest and Climate Change, 2007. Tiger Conservation Foundation Guidelines. Available at: https://ntca.gov.in/assets/uploads/guidelines/TCF_Guidelines.pdf [last accessed on 13. 05. 2024; 14:40 CET].
16.	Integrated Landscape Management Plan – Panna Tiger Reserve	Wildlife Institute of India, 2022. Integrated Landscape Management Plan for Greater Panna Landscape at a glance. Available at: https://wii.gov.in/images/images/documents/publications/rr_2022_integrated_landscape_management_plan.pdf [last accessed on 14. 05. 2024; 11:30 CET].
17.	Court Judgement at the Bombay High Court	Earth Brigade Foundation and Others v the State of Maharashtra. Bombay High Court. Case No. WP/5792/2018. Case details available at: https://bombayhighcourt.nic.in/generatenewauth.php?bhcpar=cGF0aD0uL3dyaXRlcmVhZGRhdGEvZGF0YS9uYWdjaXZpbC8yMDE4LyZmbmFtZT1XUDIwODQ2MTgwNjA5MTgucGRmJnNtZmxhZz1OJnJqdWRkYXRlPSZ1cGxvYWRkdD0wNy8wOS8yMDE4JnNwYXNzcGhyYXNlPTA5MTAyNTIyMDc1Ng== [last assessed on 09.10.2025].
18.	Court Judgement at the Uttarakhand High Court	*Suo Motu* case v Uttarakhand State Forest Department. Uttarakhand High Court. Case No. WPPIL/218/2023. Available at: https://hcservices.ecourts.gov.in/ecourtindiaHC/cases/display_pdf.php?filename=tuqye3PhFs%2BBDn75ghiOpEIoY4Q1hiLsi%2FiYfLgkCsMcf2Yh5m9ZpUsMTSUEhqOx&caseno=WPPIL/218/2023&cCode=1&cino=UKHC010199172023&state_code=15&appFlag= [last assessed on 09.10.2025; 18:30 CET]

19.	Court Judgement at the Supreme Court of India	Jerryl Avinash Banait v The State of Maharashtra. Supreme Court of India. Case No. SLP(C)/025342/2018. Available at: https://api.sci.gov.in/supremecourt/2018/33454/33454_2018_Order_11-Sep-2018.pdf [last assessed on 09.10.2025; 18:00 CET].

b. Germany

S. no.	Name of the document	Reference
1.	Modification in Bern Convention, 2025	Council of Europe, 2025. Modification of wolf protection under the Bern Convention enters into force. Available at: https://www.coe.int/en/web/portal/-/modification-of-wolf-protection-under-the-bern-convention-enters-into-force [last assessed on 19.03.2025; 08:10 CET].
	Proposal document for protection status of wolf	European Commission, 2025. Proposal for a DIRECTIVE OF THE EUROPEAN PARLIAMENT AND OF THE COUNCIL amending Council Directive 92/43/EEC as regards the protection status of the wolf (Canis lupus). File number: 2025/0058 (COD). Available at: https://www.europarl.europa.eu/RegData/docs_autres_institutions/commission_europeenne/com/2025/0106/COM_COM(2025)0106_EN.pdf (last accessed on 28.05.2025; 16:35 CET).
2.	Habitats Directive recent amendment	European Parliament and the Council, 2025. Directive (EU) 2025/1237 of 17 June 2025 amending Council Directive 92/43/EEC as regards the protection status of the wolf (*Canis lupus*). *Official Journal of the European Union*, L, 2025/1237. Available at: https://eur-lex.europa.eu/legal-content/EN/TXT/HTML/?uri=OJ:L_202501237 [last accessed on 13. 10. 2025, 13:00 CET].
3.	Guidance document – EU Commission	European Commission, 2021. "Guidance document on the strict protection of animal species of Community interest under the Habitats Directive 2021/C496/01. Available at: https://eur-lex.europa.eu/legal-

		content/EN/TXT/?uri=uriserv%3AOJ.C_.2021.496.01.0001.01.ENG&toc=OJ%3AC%3A2021%3A496%3AFULL [last accessed on 15.05.2025, 16:14 CET].
4.	Federal Nature Conservation Act	Federal Nature Conservation Act, 2009. Act on Nature Conservation and Landscape Management (BNatSchG) of 29 July 2009. Available at: https://www.gesetze-im-internet.de/bnatschg_2009/index.html#BJNR254210009BJNE004604123 [last accessed on 10. 10. 2025; 14:00 CET].
5.	Nature Protection Ordinance	Federal Ministry for the environment, health and consumer protection, 2013. Ordinance on the competence of the nature conservation authorities (NatSchZustV). Available at: https://faolex.fao.org/docs/pdf/ger125834.pdf [last accessed on 16. 05. 2024; 16:00 CET].
6.	Landscape Planning	German Federal Agency for Nature Conservation (BfN), 2012. Landscape Planning. The basis of sustainable landscape development. Available at: https://www.bfn.de/en/publications/leaflet/landscape-planning-basis-sustainable-landscape-development
7.	Wolf Management Plan, Lower Saxony (2010)	Ministry for the Environment and Climate Protection (MU), Lower Saxony, 2010. The wolf in Lower Saxony: Principles and Measures in Dealing With Wolf. Available at: https://www.dbb-wolf.de/wolf-management/wolf-management-of-federal-states/management-plans [last accessed on 21. 06. 2025; 10:30 CET].
8.	Wolf Management Plan, Lower Saxony (2022)	Ministry for the Environment, Energy, Building and Climate Protection (MU), Lower Saxony, 2022. Lower Saxony Wolf Management Plan: Principles and Guidelines for Dealing with Wild Wolves. Available at: https://www.dbb-wolf.de/wolf-management/wolf-management-of-federal-states/management-plans [last accessed on 21. 06. 2025; 10:30 CET].
9.	State hunting Act, 2022 and	Ministry of Food, Agriculture, and Consumer Protection (ML), 2022. Niedersächsischen Jagdgesetzes (NjagdG). Available at: https://voris.wolterskluwer-online.de/browse/source/csh-da-

	further amendments	filter%21a52e918e-8a02-41f8-8b62-1c4b6a92ff6a--WKDE_LTR_0000003520%23405d001a669930e1908dd76da21fd0e5 [last accessed on: 28.06.2025; 15:22 CET].
10.	Wolf Management Plan, Brandenburg, 2013 - 2017	Ministry for Rural Development, Environment and Agriculture of the State of Brandenburg, 2013. Wolf Management Plan, Brandenburg, 2013. Available at: https://www.dbb-wolf.de/wolf-management/wolf-management-of-federal-states/management-plans [last accessed on 21. 06. 2025; 10:30 CET].
11.	Wolf Management Plan, Brandenburg, 2019	Ministry for Rural Development, Environment and Agriculture of the State of Brandenburg, 2019. Wolf Management Plan, Brandenburg, 2019. Available at: https://www.dbb-wolf.de/wolf-management/wolf-management-of-federal-states/management-plans [last accessed on 21. 06. 2025; 10:30 CET].
12.	State Hunting Act, 2003 and further amendments	Ministry of Agriculture, Food Industry, Environment and Consumer Protection (MLUK), 2023. Jagdgesetz für das Land Brandenburg (BbgJagdG). https://bravors.brandenburg.de/gesetze/bbgjagdg [last accessed on 28.06.2025; 15:14 CET].
13.	Guidance document on the protection of animal species	European Commission, 2007. Guidance document on the strict protection of animal species of Community interest under the Habitats Directive. Available at: https://op.europa.eu/en/publication-detail/-/publication/1059d053-7082-421a-9bdc-54b2749c16c7 [last accessed on 27. 07. 2024; 10:00 CET].
14.	*Praxisleitfaden* (translates to "practical guide")	Federal Ministry for Environment, Nature Conservation and Nuclear Safety, 2021. Practical guidelines for issuing species protection permits: Exceptions according to §§ 45 and 45a BNatSchG in the case of wolves, especially in the case of livestock kills: Practice-oriented test sequence and test content based on the current legal basis. Available at: https://www.bmuv.de/download/praxisleitfaden-wolf [last accessed on 21. 06. 2024; 14:00].
15.	Bold Wolves	Reinhardt, I., Kaczensky, P., Frank, J., Knauer, F. and Kluth, G., 2020. How to deal with bold wolves. *Recommendations of the DBBW. BfN-*

		Skripten, *577*. Available at: https://revolutionrov.org/wp-content/uploads/2022/05/637423653359535374_reinhardt_rep_bold_wolves.pdf [last accessed on 13. 11. 2024; 10:00 CET].
16.	Wolf Directive, 2022	Lower Saxony State Agency for Water Management, Coastal Defence and Nature Conservation, 2020. Richtlinie über die Gewährung von Billigkeitsleistungen und Zuwendungen zur Minderung oder Vermeidung von durch den Wolf verursachten wirtschaftlichen Belastungen in Niedersachsen (Richtlinie Wolf). Available at: https://wolfcenter.de/wp-content/uploads/2021/04/Richtlinie_Wolf_incl_Anlagen1u2_Jan2020.pdf [last accessed on 21.07.2024; 13:00 CET].
17.	Guide to the inclusion of the Wolf in Hunting Law, 2022	Action Alliance Forum Nature (AFN), 2022. Wolf management: Proposal for action for a practice-oriented wolf management in the German cultural landscape. Available at: https://www.bauernverband.de/fileadmin/user_upload/dbv/themendossiers/Wolf/WbW-3teAuflage-Wolfsmanagement-26.01.2022.pdf [last accessed on 12.05.2024; 19:00].
18.	Draft of law on wolf management	German Bundestag, 2019. Draft of a law on wolf management (D. no. 19/10792). Available at: https://dserver.bundestag.de/btd/19/107/1910792.pdf [last accessed on 28.01.2025; 15:33 CET].
19.	Draft of law on wolf management	German Bundestag, 2019. Resolution recommendation and report of the Committee on Food and Agriculture (10th Committee) (D. no. 19/16147). Available at: https://dserver.bundestag.de/btd/19/161/1916147.pdf [last accessed on 24.01.2025; 12:33 CET].
20.	Draft to amend the BNatSchG	German Bundestag, 2019. Draft law of the Federal Government. Draft of a second law to amend the Federal Nature Conservation Act (D. no. 19/10899). Available at: https://dserver.bundestag.de/btd/19/108/1910899.pdf [last accessed on 28.01.2025; 16:21 CET].

21.	Draft of a Second Act to Amend the BNatSchG – Statement by the Federal Council and response by the Federal Government	German Bundestag, 2019. Information from the Federal Government. Draft of a second law to amend the Federal Nature Conservation Act (D. no. 19/13289). Available at: https://dserver.bundestag.de/btd/19/132/1913289.pdf [last accessed on 28.01.2025; 15:46 CET].
22.	Court Judgement at the CJEU	Asociación para la Conservación y Estudio del Lobo Ibérico (ASCEL) v Administración de la Comunidad de Castilla y León. Court of Justice of the European Union. Case No. - C-436/22. Available at: https://eur-lex.europa.eu/legal-content/EN/TXT/HTML/?uri=CELEX:62022CJ0436&qid=1760026843114 (last accessed on 29.05.2025; 14:00 CET).
23.	Court Judgement at the CJEU	Umweltverband WWF Österreich and Others v Tiroler Landesregierung. Court of Justice of the European Union. Case No. - C-601/22. Available at: https://eur-lex.europa.eu/legal-content/EN/TXT/HTML/?uri=CELEX:62022CJ0601&qid=1760026906196 (last accessed on 30.05.2025; 11:00 CET).
24.	Court Judgement at the CJEU	Luonnonsuojeluyhdistys Tapiola Pohjois-Savo – Kainuu ry v Risto Mustonen and Others. Court of Justice of the European Union. Case number - C-674/17. Available at: https://eur-lex.europa.eu/legal-content/EN/TXT/HTML/?uri=CELEX:62017CJ0674&qid=1760026437491 (last accessed on 03.06.2025; 12:30 CET).
25.	Court Judgement at the CJEU	Commission of the European Communities v Republic of Finland. Case number - C-342/05. Court of Justice of the European Union. Available at: https://eur-lex.europa.eu/legal-content/EN/TXT/HTML/?uri=CELEX:62005CJ0342&qid=1760026628824 (last accessed on 04.06.2025; 17:00 CET).

S4: Laws that define when a tiger can be captured or killed for the case of tigers in India and when a wolf/wolf pack can be declared as problem individual(s) to be killed in Germany.

Country	Definition of problematic individual	Responsible authority for decision-making and laws
India	Based on a Standard Operating Protocol (SoP) prepared by the NTCA, a tiger is considered 'dangerous to human life" 1. When it has actively predated on a human, i.e. it stalks and hunts to eat human flesh and after hunting eats the body. The SoP recommends the CWLW to declare the individual without doubt as 'dangerous to human lives'. In a layman's language, such an animal is declared a 'man-eater' although the term is not used officially. Not all cases of tiger attacks on humans are intended attacks, i.e. are a result of accidents due to sudden encounters with humans or when a tiger could mistakenly kill a human confusing it to be a prey species.<br><br>2. If it is disabled or deceased as to be beyond recovery.<br><br>However, according to the Wildlife Protection Act, 1972 apart from killing, capturing animals is also defined as hunting (§2(16)(a) and (b)). Hence, in this case,	• Under the §11(1a) of the WPA, 1972, the 'Chief Wildlife Warden' (CWLW), a state officer, permits a person to lethally control a schedule I species, in the context of this study, a tiger individual, if the animal cannot be captured, tranquilized or translocated and, unless the animal cannot be rehabilitated in the wild. Hence, she/he provides a decision in writing on whether to rehabilitate the animal in the wild or in extreme cases relocate the individual to a zoo or rescue center, or when attempts to capture the individual have failed, to kill the individual.<br>• Under §11(2) the WPA, 1972 provide provision to a common person to kill a tiger in self-defense or to protect another person.

	3. When it has repeatedly killed livestock in surrounding villages.<br><br>4. When a tiger has entered human settlement and needs to be tranquilized for capture and be translocated to the nearest tiger reserve.<br><br>5. Can be killed by a common person in self-defense or to protect another person.	
Germany*	1. If a wolf individual shows brazen behavior that may endanger people.<br><br>2. Wolves that have learnt crossing electric fences (that were properly installed) that are meant to protect livestock, as repetitive undesirable behavior of killing livestock may lead to increased negative perception in people towards wolves.<br><br>3. Wolves that have killed/injured livestock in open grazing areas that can't be fenced.<br><br>4. Wolf-dog hybrids after the first four generations of hybridization. However, there are cases of removal of hybrids from the wild based on the information	• Article 45(a), Para 4, in case of wolf attacks on livestock, a nature conservation authority after examination of the case, provides an individual decision of killing an individual or an entire pack.

	of breeding between a wolf and a dog, also evident from the phenotypic characteristics.	

* Although the Bern Convention and Habitats Directive reclassified the wolf from 'strictly protected' to 'protected' in March 2025, Germany had not amended the relevant provisions of the BNatSchG (national legislation) at the time of manuscript submission; thus, the regulations presented in the table remain applicable.

S5: Details of working rules, as well as laws that determine the 'rules-in-use' within the action arena for our study cases for managing human-tiger conflict in India and human-wolf conflict in Germany with lethal control.

Rules-in-use	*India*	*Germany*	*Reference*
Boundary rules - What laws protect the carnivores in respective countries	Tiger strictly protected as a Schedule I species under the WPA, 1972.	Wolves are protected under the Habitats Directive and the BNatSchG (Federal Nature Conservation law).	Wildlife Protection Act (WPA), 1972, and Amendment Acts of 2006 and 2023.<br><br>Habitats Directive (Annex V); BNatSchG §45 and 45(a).
Scope rules - When can a carnivore be allowed to be killed to manage "action situation" in both countries?	When a tiger has killed multiple humans and is declared 'dangerous to human lives'. Killing a tiger is a last resort as per the WPA, 1972, whereby attempts need to be made to capture the individual alive and translocate it to a zoo / rescue centre, if it cannot be rehabilitated. Since the act of capturing the animal is considered hunting, we do not consider other cases where a tiger needs to be captured for relocation or translocation.	1. When a wolf/ a wolf pack has shown 'bold behaviour' by repeatedly approaching human / human settlements too closely (less than 30-metre distance).<br>2. When a wolf/ a wolf pack has repeatedly killed livestock and/or has learnt to overcome preventive measures (electric fencing, livestock guarding dogs).<br>3. In case damage to livestock / threat to human life / bold behaviour cannot be attributed to a wolf / wolf pack, killing of a wolf pack in the close	WPA, 1972; NTCA 2007.<br><br>Brandenburg Wolf Management Plan (2019); Wolf *Ordinance* (Brandenburg) (2022); Lower Saxony Wolf Management Plan (2022); Lower Saxony Wolf *Ordinance* (2020); 45(a), Para (2) and (3) BNatSchG.

		proximity of the place of damage events is allowed until an absence of the damage is observed.<br>4. A wolf-dog hybrid can be killed.	
Position rules - Who decides on individual cases to permit lethal measures?	The WPA, 1972 provides the CWLW of a State with the power to solely decide on whether or not to declare a tiger 'dangerous to human lives', specifying killing an animal as the last resort.	Depending on the state rules the agency for the environment ministry or the ministry itself is responsible to make decisions on declaring a wolf as a problem individual.	§11(1a) of the WPA, 1972; Expert interviews [IN_001; IN_007].<br><br>Expert interviews [DE_010; DE_013].
Aggregation rules – What is the mechanism fir providing the information?	In India, teams such as Rapid Response Teams (RRTs) (names may vary from State to State, but their composition and responsibilities are similar; number of people in the teams may vary between regions based on the frequency of conflicts) are responsible to assist as a front-line in conflict cases. A part of the team from the forest department is responsible to identify the tiger, another part of the staff works with the RRTs, to monitor village to prevent	A team of inspectors from the responsible ministry of the state is responsible to examine the site of presumable wolf attack. The team checks if the livestock were fenced properly (to confirm that the wolf has learnt how to cross fences or not). DNA samples from the attacked livestock is sent to lab for confirmation. Based on the DNA reports, and on-site inspection, the team prepares a report on	NTCA 2007; NTCA 2013; Expert interviews [IN_001; IN_007].<br><br>Expert interviews [DE_004; DE_010; DE_022].

	further attacks and manage crowd to ensure the safety of people and staff during the event of capturing of a tiger. A Committee involving experts, local NGOs, veterinarian and a member from village *Panchayat* (council) provides technical support to the DFO/FD/DD of the nearest protected area in managing the site of conflict.	the case, which is then submitted to the responsible agency.	
Information rules – What information is used for decision-making?	A Field Director (FD) / Deputy Director (DD) in the case of a tiger reserve, or a Divisional Forest Officer (DFO) informs the CWLW with on-ground situation. They also prepare a report to support the CWLW for their decision. The CWLW may enquire for more information, if necessary, to make their decision. The CWLW may refer to guidelines prepared by the NTCA to make their decisions. A tiger is identified based on data repository of the Individual tiger Identification Database (ITID), which allows	Genetic information determines whether or not the wolf in question was responsible for more attacks in the past (based on genetic information of the wolves compiled in a data repository by the Senckenberg institute, Germany). This information, in addition to a report prepared from the site of incident, (aggregation rules) act as the sources of information for the decision-maker authority.	NTCA 2007; NTCA 2013; Expert interviews [IN_001; IN_003; IN_008].<br><br>Expert interviews [DE_004; DE_006; DE_010; DE_014].

	the forest department to know about its territorial information and confirm the killings.		
Choice rules – What determines the choice of whether or not kill a carnivore?	Ideally, when all reports received from the ground situation is received and the CWLW is satisfied that the tiger has become ‘dangerous to human lives’, they may declare capturing of the tiger individual, and in extreme situation when the tiger cannot be captured after multiple attempts, while cases of attacks on humans continue, they may order killing the individual. However, trade-offs play a role in certain cases arising from crossed thresholds of tolerance in a society.	Ideally, based on the reports presented by the responsible agencies, a decision-maker may allow killing a wolf or a wolf pack that has been declared ‘problematic’ or ‘bold’. However, their decisions may be influenced by trade-offs arising from vote-bank politics.	WPA, 1972; Expert interviews [IN_001; IN_009; IN_012].<br><br>Brandenburg Wolf *Ordinance*; Lower Saxony Wolf *Ordinance*; Expert interviews [DE_003; DE_004; DE_011].
